# Fraternine, a Novel Wasp Peptide, Protects against Motor Impairments in 6-OHDA Model of Parkinsonism

**DOI:** 10.3390/toxins12090550

**Published:** 2020-08-27

**Authors:** Andréia Mayer Biolchi, Danilo Gustavo Rodrigues de Oliveira, Henrique de Oliveira Amaral, Gabriel Avohay Alves Campos, Jacqueline Coimbra Gonçalves, Adolfo Carlos Barros de Souza, Marcos Robalinho Lima, Luciano Paulino Silva, Márcia Renata Mortari

**Affiliations:** 1 Laboratory of Neuropharmacology, Department of Physiological Sciences, University of Brasília, Brasília, DF 70910-900, Brazil; andreia.biolchi@gmail.com (A.M.B.); hoamaral.29@gmail.com (H.d.O.A.); gabriel_avohay@hotmail.com (G.A.A.C.); jacq.coimbra@gmail.com (J.C.G.); adolfo_quimica@hotmail.com (A.C.B.d.S.); robalinho.lima@gmail.com (M.R.L.); mmortari@unb.br (M.R.M.); 2 Laboratory of Evolutionary Ecology & Conservation, Department of Animal and Plant Biology, State University of Londrina, Londrina, PR 86051-970, Brazil; 3 Laboratory of Mass Spectrometry, Embrapa Genetic Resources and Biotechnology, Brasília, DF 70770917, Brazil; luciano.paulino@embrapa.br

**Keywords:** wasp venom, antiparkinsonian activity, motor behavior, Parkinson’s disease, neuroprotective effect

## Abstract

Parkinson’s disease (PD) is a progressive neurodegenerative condition that affects the Central Nervous System (CNS). Insect venoms show high molecular variability and selectivity in the CNS of mammals and present potential for the development of new drugs for the treatment of PD. In this study, we isolated and identified a component of the venom of the social wasp *Parachartergus fraternus* and evaluated its neuroprotective activity in the murine model of PD. For this purpose, the venom was filtered and separated through HPLC; fractions were analyzed through mass spectrometry and the active fraction was identified as a novel peptide, called Fraternine. We performed two behavioral tests to evaluate motor discoordination, as well as an apomorphine-induced rotation test. We also conducted an immunohistochemical assay to assess protection in TH+ neurons in the *Substantia Nigra* (SN) region. Group treated with 10 μg/animal of Fraternine remained longer in the rotarod compared to the lesioned group. In the apomorphine test, Fraternine decreased the number of rotations between treatments. This dose also inhibited dopaminergic neuronal loss, as indicated by immunohistochemical analysis. This study identified a novel peptide able to prevent the death of dopaminergic neurons of the SN and recover motor deficit in a 6-OHDA-induced murine model of PD.

## 1. Introduction

Parkinson’s disease (PD) is a neurodegenerative multisystem disorder. However, its initial clinical manifestations are mainly associated with the motor system, and they consist of: resting tremor, rigidity, bradykinesia and postural instability [1]. These manifestations result directly from a massive depletion of dopaminergic neurons in the nigrostriatal pathway, consequently leading to a reduction in dopamine neurotransmission, which is considered the main neuropathological feature of the disease [2]. Non-motor symptoms manifest as depression, autonomic disorders, cognitive dysfunction, sleep disorders and dementia [3,4]. Etiopathogenic hypotheses characterize PD as the combination of several factors such as the action of environmental neurotoxins, protein misfolding and aggregation, impairment of protein clearance pathways, mitochondrial abnormalities, brain aging, genetic predisposition and gut microbiota [5,6,7].

Despite extensive research, the current commercially available drugs for the treatment of PD only present symptomatic efficacy, and their use is incapable of halting the deterioration of dopaminergic neurons. As a result, it is highly necessary to study and develop new drugs that may prevent the progression of the disease and neuronal loss [8]. In addition, significant health costs associated with the treatment of PD have resulted in the urgency of finding new therapies for the treatment or prevention of this disease [9]. In this context, compounds isolated from animal venoms have gained great interest in the screening of new therapeutic molecules for the treatment of neurological disorders [10]. The vast repertoire of animal venom molecules have evolved as tools for predation and defense, which have, interestingly, gained the ability to interact directly with vital physiological and biochemical processes into the nervous and cardiovascular systems [11,12]. In particular, naturally occurring peptides have brought great interest as platforms for drug development [13]. The peptide Exendin-4, isolated from the venom of *Heloderma suspectum*, known as the Gila monster, has demonstrated neuroprotective effects in different parkinsonian models [14,15,16]. Additionally, previous studies have demonstrated that crude bee venom (BV) from *Apis mellifera,* associated with acupuncture, has also appeared to be a promising compound that interferes with the progression of PD [17,18].

The venom of Epiponini social wasps is typically composed of four kinds of peptides: wasp kinins, chemotactic peptides, mastoparans and other peptides [19,20]. Although the first three kinds are known toxic molecules, a number of peptides isolated from the venom of social wasps were found to exert a range of pharmacological effects, including: antinociceptive, anxiolytic, anti-inflammatory, and antiepileptic, showing a broad potential for therapeutic applicability [21,22,23,24]. Moreover, this knowledge of the potential pharmacological aspects of these peptides will shed light on the study and development of new drug candidates for the treatment of neurological disorders, like PD.

In this study, we tested the potential neuroprotective action of a novel peptide—Fraternine—isolated from the venom of the social wasp *Parachartergus fraternus*, using the 6-hydroxydopamine (6-OHDA)-induced PD mouse model by striatal infusion.

## 2. Results

### 2.1. Purification and Structural Analysis

**Figure 1:**
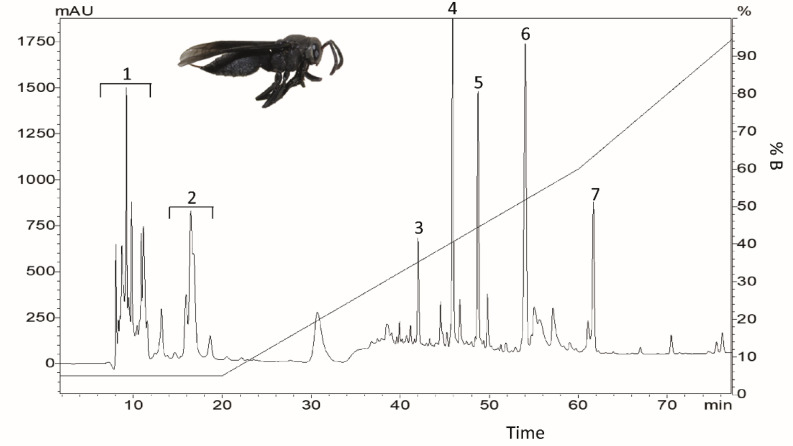
Chromatographic profile of the ultrafiltered venom of *Parachartegus fraternus* in a semi preparative column C18 Phenomenex. ACN gradient is shown in the dark line. Numbers represent fractions of the venom that were collected for further analysis. Fraction 6 corresponds to the compound denominated Fraternine.

Approximately 1000 glands and reservoirs were extracted, which resulted in 208.8 mg of ultrafiltered venom after maceration and ultrafiltration. The ultrafiltered material underwent reverse-phase HPLC fractionation. Thirty-four chromatographies were made, total time of the run was 100 min, and the fractions were eluted until 75 min; the more abundant peaks were divided into 7 fractions (Figure 1).

The chromatogram was similar to that obtained using the venom of the same species [25] and closely related ones [19,20,26], in which the first two fractions were formed by biogenic amines and neurotransmitters, like serotonins. As the objective of the work was to detect new peptides, fractions 1 and 2 were not tested. After analysis by mass spectrometry of the fractions, it could be seen that the peptides were eluted after 30 min.

After separating the compounds by HPLC, the fractions used in the screening were sent to the MALDI-TOF mass spectrometer to determine the molecular masses and evaluate the purity. Fraction 3 presented the three most abundant peptides of monoisotopic masses [M + H]^+^ = 1055.6 (major), [M + H]^+^ = 1358.8 and [M + H]^+^ = 1479.9. Fraction 4 showed a high purity level with an intense signal of [M + H]^+^ = 1358.8; the presence of this mass in fraction 3 is probably derived from some contamination. Fraction 5 is formed by a peptide with monoisotopic mass [M + H]^+^ = 1209.9. Mass data revealed that fraction 6 showed a single monoisotopic mass of [M + H]^+^ = 2748.5; due to the absence of other contaminants, the fraction was not subjected to other chromatography steps. Mass spectra with Na^+^ and K^+^ adducts were also observed (Figure S1a,b). The mass spectrum of fraction 7 revealed the presence of a predominant compound of mass [M + H]^+^ = 1566.9, its adducts with sodium [M + Na]^+^ = 1588.9 and potassium [M + K]^+^ = 1604.9. De novo Sequencing revealed that this peptide is Agelaia-MP, which was not evaluated in this work due to its characteristics of the mastoparan class (Table 1).

After an initial screening in the 6-OHDA model of parkinsonism with the fractions (data not shown) and due to a greater abundance, fraction 6 was selected for sequencing and underwent further tests, and the peptide was named Fraternine. In total, 6 mg of the peptide was purified, and its high degree of purity was confirmed by chromatography and mass spectrometry. The sequence of Fraternine was determined by de novo sequencing based on mass spectral analysis. However, analyses of MS/MS fragmentation experiments made it possible to obtain only the partial amino acid sequence of the peptide (Figure S2). Therefore, we undertook the process of reducing peptide bonds with dithiothreitol (DTT) and later cysteine alkylation with iodoacetamide (Figure S3). After peptide reduction, enzymatic hydrolyses were performed with Glu-C (Figures S4 and S5) and immobilized trypsin (Figures S6 and S7). As showed in Figure S2 and Table 2, the final sequence was confirmed by the analysis of two series of ions, the -b and -y, and fragment analysis after enzymatic hydrolases. Finally, it resulted in the sequence:

Fraternine: Ile/Leu-Ser-Phe-Gln-Gln-Val-Lys-Glu-Lys-Val-Cys-Lys-Val-Glu-Ala-Lys-Ile/Leu-Gly-Lys-Lys- Ile/Leu-Pro-Phe-Cys-NH2 ([M + H]^+^ = 2748.5 Da).

**Figure 2:**
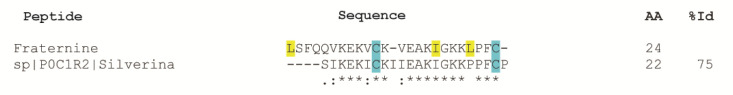
Alignment with peptide from wasp venom. Toxins are presented by their UniProt KB codes or name. Capital letters denote amino acids. (*) represents identical amino acid residue. (:) designates a conservative modification in the residue of amino acid. (.) means semi-conservative modification. The white space represents an absence of identity between amino acid residues. Cys residues shaded in blue are connected by a disulfide bond. Ambiguities of isoleucine and leucine are shaded in yellow. AA means amino acid residues, and %Id is the percentage of sequence identity with Fraternine.

Because of the similarity displayed to the peptides that were isolated from social wasps’ venom and structurally characterized with disulfide bonds, and upon comparison with data from the protein library (Blastp), it can be seen that this is a new peptide. Using the Blastp, the primary sequence of Fraternine showed a high percentage of identity (75%) with the peptide Sylverin isolated from the venom of the *Protonectarina sylveirae* wasp (Figure 2). Peptides of the Sylverin group contain from 21 to 25 amino acid residues in their primary sequences, presenting an intramolecular single disulfide bridge, and are potent mast cell degranulators similar to mastoparan peptides, but without hemolytic activity [29].

### 2.2. Evaluation of the Fraternine Peptide in the Murine Model of PD

The motor coordination of the animals was evaluated on days 3, 4, and 5 after the injury. In each test, the animals were put on the rotarod equipment 30 min after the administration of treatments, and a reading of the latency to fall was made.

**Figure 3:**
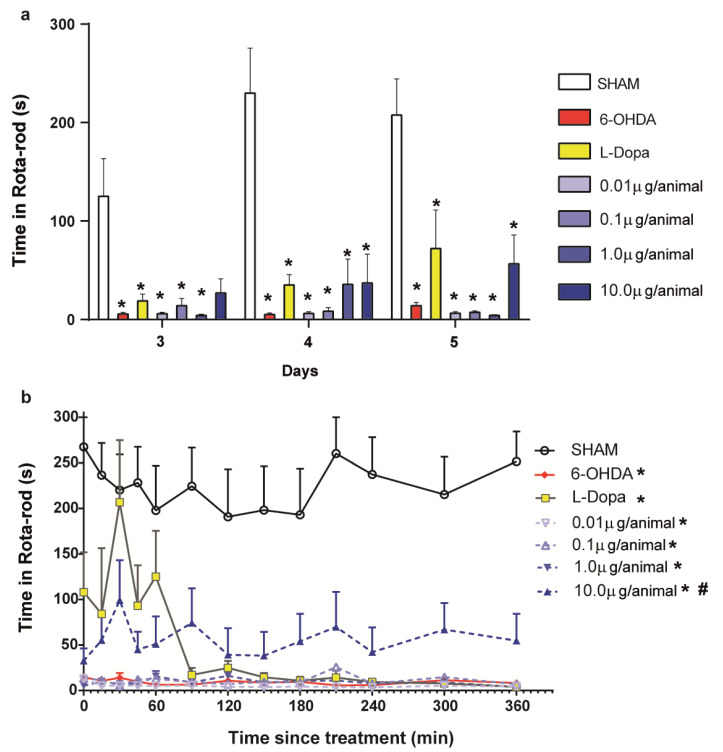
Motor coordination in the Rota-rod of mice SHAM (*n* = 5) or 6-OHDA lesioned and treated with vehicle (6-OHDA, *n* = 7), L-DOPA + benserazide (6 mg/Kg and 5 mg/kg, respectively) via i.p. (L-Dopa, *n* = 6), Fraternine 0.01 µg/animal (*n* = 7), Fraternine 0.1 µg/animal (*n* = 5), Fraternine 1.0 µg/animal (*n* = 7) or Fraternine 10 μg/animal (*n* = 6). (**a**) Performance of mice in the rotarod test on 3 consecutive days (days 3, 4 and 5). (**b**) Performance of mice in the R-TET, which was executed at 0, 15, 30, 45, 60, 90, 120, 150, 180, 210, 240, 300, 360 min after the treatments on day 5. The symbol * represents significant difference (*p* < 0.05) with SHAM group and # represents difference (*p* < 0.05) with 6-OHDA group.

Fraternine, via i.c.v., induced a dose-dependent antiparkinsonian effect in the Rotarod test. The two-way analysis of variance indicated that the interaction between time since the 6-OHDA lesion and treatment was not significant [F (12, 58) = 1.22; *p* = 0.84]. We also found that the time since the 6-OHDA lesion did not affect the coordination of mice on the rotarod [F (2, 33) = 2.82; *p* = 0.07]. However, we found that the coordination of mice on the rotarod was affected by treatment [F (6, 29) = 17.04; *p* < 0.0001]. The Bonferroni post-hoc test revealed significant differences among the three treatment times. At days 4 and 5, all groups treated with the peptide (*p* < 0.001), L-DOPA (*p* < 0.01) and 6-OHDA group (*p* < 0.001) showed a significant difference from the saline group SHAM. On day 3, the group 6-OHDA (*p* = 0.010), L-DOPA (*p* = 0.0488) and peptide dosages of 0.01 (*p* = 0.011), 0.1 (*p* = 0.028) and 1.0/animal (*p* = 0.009) showed a significant difference from the control group, but the higher dose of 10.0 μg/animal (*p* = 0.064) did not, revealing a dose-dependent effect (Figure 3a).

On the fifth day after injury, the animals were evaluated on the R-TET for 6 h, where we found a significant difference between the different treatments [Linear mixed model: F (6, 42) = 20.02; *p* < 0.001]. Animals treated with the 10.0 μg/animal dose spent less time on the rotarod compared with the SHAM group [*p* < 0.001]. However, these animals were capable of spending a longer amount of time on the rotarod when compared to the animals in the 6-OHDA group [*p* = 0.013]. Furthermore, animals treated with L-DOPA and 10.0 μg/animal of Fraternine spent a similar amount of time on the rotarod during the R-TET (*p* > 0.05). This indicates that these treatments have similar effects in animal coordination on the rotarod. However, after 60 min, animals treated with L-DOPA presented a similar amount of time on the rotarod to those treated with 6-OHDA, which indicates the wearing down of L-DOPA effects with time (Figure 3b). Conversely, animals treated with 10.0 μg/animal of Fraternine showed a constant effect on their coordination during the 6-hour period, which indicates that Fraternine may have protected the dopaminergic neurons from death, allowing their function to be maintained and, thus, an improvement in motor performance (Figure 3b). This dose also showed similarity with the SHAM group at 30 min (*p* > 0.05), demonstrating a reduction in the motor deficits. The peptide was also evaluated for its potential toxic motor effect on the amount of time the animals spent on the rotarod by treating animals with Fraternine without induction of parkinsonism. The linear mixed model revealed significant differences for time [F (12, 252) = 3.15; *p* < 0.001] and treatment [F (2, 12) = 145.36; *p* < 0.001], revealing no difference from Sham control (*p* = 0.62) and better motor activity than the toxic 6-OHDA (*p* < 0.001) (Figure S8). This result shows that Fraternine does not cause motor alterations.

### 2.3. Apomorphine-Induced Turning Behavior

**Figure 4:**
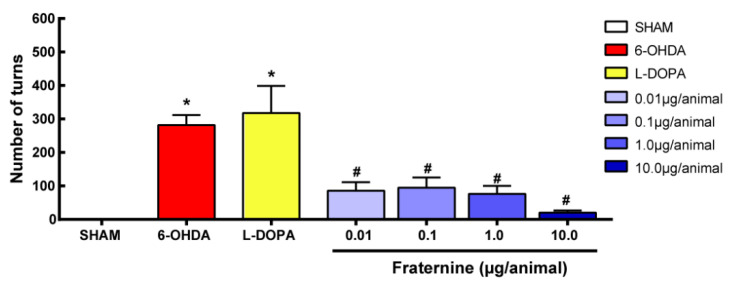
Number of turns exhibited on the apomorphine-induced turning behavior test, which is a dopamine agonist that was injected via s.c. (5 µg/animal) on day 7. Mice were divided into the following groups: SHAM (*n* = 5) or 6-OHDA lesioned and treated with vehicle (6-OHDA, *n*= 10), L-DOPA + benserazide (L-Dopa, *n* = 8), Fraternine 0.01 µg/animal (*n* = 8), Fraternine 0.1 µg/animal (*n* = 9), Fraternine 1.0 µg/animal (*n* = 8) or Fraternine 10 μg/animal (*n* = 9). The symbol * represents difference (*p* < 0.05) with SHAM and # represents difference (*p* < 0.05) with 6-OHDA.

On day 7 after the 6-OHDA injury, animals were injected subcutaneously with apomorphine, and the total number of induced rotations was quantified for all groups. This test provides an evaluation of the extent to which dopaminergic neurons are depleted in hemiparkinsonian models, allowing an assessment of behavior regarding peptides’ neuroprotective effect. We observed a marked contralateral rotation in both 6-OHDA control mice treated with saline and L-Dopa. One-way ANOVA showed a significant difference in the number of contralateral rotations between treatments [F (7, 45) = 8.87; *p* < 0.0001]. The SHAM group showed no rotations, while animals treated with 6-OHDA showed higher numbers of contralateral rotations. The animals treated with different doses of the Fraternine peptide showed a smaller number of rotations compared with 6-OHDA and L-DOPA group (*p* < 0.001) and showed no significant differences from the SHAM group (Figure 4). Therefore, Fraternine prevented apomorphine-induced rotation in the 6-OHDA hemiparkinsonian model, with the highest dose being the most effective.

### 2.4. Quantification of Dopaminergic Neurons in Substantia Nigra

**Figure 5:**
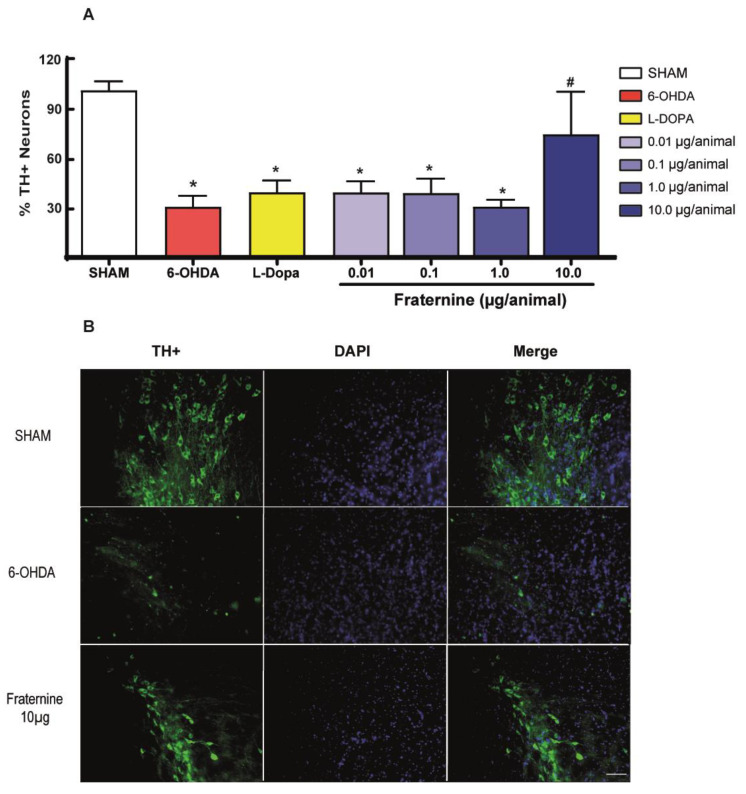
(**A**) Percentage of neurons reactive for tyrosine-hydroxylase in immunohistochemistry compared to the unlesioned side of the brain of mice that were SHAM (*n* = 7) or 6-OHDA lesioned and treated with vehicle (6-OHDA, *n* = 8), L-DOPA + benserazide (L-Dopa, *n* = 5), Fraternine 0.01 μg/animal (*n* = 3), Fraternine 0.1 µg/animal (*n* = 4), Fraternine 1.0 μg/animal (*n* = 3) or Fraternine 10 μg/animal (*n* = 3). (**B**) Photo in the microscope (200× magnification) of a section of *Substantia nigra* of mice in the groups SHAM, 6-OHDA and Fraternine 10 µg/animal stained for tyrosine hydroxylase (TH^+^-green) and DAPI (blue). The white scale bar in the right corner of the image represents 50 µm. The symbol * represents difference (*p* < 0.05) with SHAM and # represents difference (*p* < 0.05) with 6-OHDA.

In order to count the remaining dopaminergic neurons in SN, we performed an immunohistochemical assay to stain tyrosine hydroxylase reactive (TH+) neurons. Since we applied a hemi-parkinsonian model, we considered the contralateral hemisphere of the mouse brain (right hemisphere), which did not receive 6-OHDA, as a healthy control, and compared both sides to analyze the percentage of DA neuron degeneration. Therefore, the i.c.v. injection of Fraternine in the higher dose (10.0 μg/animal) significantly protected neuronal death induced by 6-OHDA, preserving about 74.1% of the dopaminergic neurons. The ANOVA (one-way) revealed a significant difference between the percentage of TH+ neurons in the different treatments [F(6, 26) = 15.08; *p* < 0.001]. The 6-OHDA group presented a significant reduction in the number of neurons in SN, in comparison to the SHAM group (*p* < 0.01), losing about 70.7% of dopaminergic neurons. SHAM also showed a significant difference with L-DOPA and the doses 0.01, 0.1 and 1.0 µg/animal of the peptide (*p* < 0.05), showing that the treatment with L-DOPA and the smaller doses of the peptide did not protect the neurons. The lowest dose (0.01 μg/animal) protected about 38.9% of TH+ neurons, while the intermediate doses of 0.1 and 1 μg/animal protected about 38.5% and 30.2% respectively. (Figure 5).

## 3. Discussion

In the present study, we identified a novel natural peptide that managed to protect DA neurons in the SN and improve the motor performance of animals induced with parkinsonism via 6-OHDA. Therefore, Fraternine exhibited a potential antiparkinsonian effect. Changes in the development and design of new drugs for the treatment of PD have occurred in the last decades, with the purpose of halting the progression of the disease [30]. According to Youdim [8], drugs that act on neurodegeneration can be divided into three categories: neuroprotection, neurorestoration and neurorescue. Neuroprotection is defined when the drug slows down or stops the progression of the disease; the neurorestoration drug replaces non-viable cells with viable cells, restoring neuronal damage; and neurorescue drugs save cells that have started the process of neuronal death [8]. However, commercial drugs available for the treatment of PD have so far only shown efficacy in the treatment of symptoms, and the deterioration of dopaminergic neurons continues. Thus, it is necessary to study and develop new medicines that can prevent the progression of the disease and prevent neuronal loss [31].

Peptides represent a unique and promising class of pharmacological compounds capable of treating neurological diseases because of their intrinsic signaling in physiological functions. In this sense, naturally occurring peptides are known to be selective and efficacious molecules, which bind to specific receptors that closely mimic natural pathways [13,32]. Thus, peptides isolated from animal venoms are a powerful tool in the study of new drugs, and the pursuit of therapies based on these compounds has evolved over time. Over 60 peptides have been approved for the market in the United States, Europe, and Japan. In addition, around 150 peptides are in active clinical development, and 260 have been tested in human clinical trials [33]. Interestingly, naturally occurring peptides have been proven to exert a range of pharmacological effects, such as neurotrophic, anti-apoptotic, anti-inflammatory and other neuroprotective activities [10].

Currently, there are more than 60 compounds isolated from animal venoms with therapeutic action approved by the FDA, and more than 500 therapeutic peptides in pre-clinical development, given that many of those are already considered as prototypes for new remedies [13]. However, an important drawback in the use of natural occurring peptides is their poor chemical and physical stability, and short circulating plasma half-life [13]. These aspects diminish the interest in their therapeutic use and must be addressed in the development of new drugs. Thus, new technologies have been emerging in order to circumvent these drawbacks. Rational design of therapeutic peptides is a way to improve chemical and physical stability under physiological conditions, by modifying specific amino acid residues or doing PEGylation to improve in vivo stability of peptides. Moreover, engineered nanomaterials (drug delivery systems), multifunctional and cell-penetrating peptides, and new technologies for alternative administration routes are applicable tools that extend the employment of naturally occurring peptides as medicines, by increasing selectivity, specificity and circulating plasma half-life.

One interesting peptide that has shown very promising outcomes for the treatment of PD is exendin-4, with several studies demonstrating its potential neuroprotective effect [10]. In fact, exendin-4 has already been addressed in clinical trials for PD treatment, and it was shown to improve patients’ motor impairment [34,35,36]. Nevertheless, considering the short circulating plasma half-life of exendin-4, calculated as an estimated value of 2.0 h, additional studies have been performed to improve chemical and physical stability of exendin-4. NLY01, a Glucagon-like peptide-1 receptor (GLP1R) agonist, is a pegylated long-acting peptide with a similar sequence to exendin-4 and extended half-life (88 h versus the 2 h half-life of exendin-4 in non-human primates). It was found that NLY01 improved motor behavior performance and reduced neuropathological features in both human A53T α-synuclein transgenic mice, and α-synuclein preformed fibril mouse model of PD [37]. Therefore, it is important to consider approaches using different technologies to improve natural peptides’ stability in the physiological environment, in order to increase interest in their clinical applicability.

The peptide isolated in this study—Fraternine—exhibited a more prominent effect on motor dysfunction relief in comparison to L-DOPA. Animals treated with Fraternine presented an overall better motor coordination behavior than parkinsonian mice. This outcome highlights Fraternine as a possibly better alternative drug for the long term. In the same test, groups treated with L-DOPA improved motor performance in the first 60 min, followed by a decline in its effect. Fraternine may have protected the dopaminergic neurons from death, allowing its function to be maintained and improving motor performance. Moreover, L-DOPA is also known to induce dyskinetic movements in patients with prolonged use of this medicine. L-DOPA is considered the gold standard treatment for PD, acting as symptomatic relief without the ability to halt disease progression [38].

Fraternine reduced the number of contralateral rotations induced by apomorphine in 6-OHDA lesioned mice. Apomorphine is a potent agonist of the D1/D2 dopamine receptors, and apomorphine-induced rotations have been shown to be one of the best measures of intensity of lesion in dopaminergic neurons of SN in hemiparkinsonian models [39]. Fraternine may either prevent cell death or activate neurogenesis to restore dead cells, both of which prevent the progression of the disease. This was corroborated by the number of neurons reactive to TH, which revealed a significantly higher percentage of neurons in the SN of the uninjured side of the brain compared to the side injured with 6-OHDA. The viability of TH-reactive neurons yields noticeable results that can be used to assess the neuroprotective capacity of the test compound. Similarly, a study by Kim and colleagues [40], in which mice were treated with *Apis mellifera* crude poison(s), reported increased TH-reactive neurons in SN compared to healthy animals. However, more results are necessary to elucidate Fraternine’s neuroprotective effects, and also to explore its possible mechanism of action.

## 4. Conclusions

Overall, we visualize Fraternine as a potential drug-model for the development of new therapies for neurological disorders, especially PD. The results suggest that Fraternine potentially triggered neuroprotective activity and improved motor coordination in a 6-OHDA-induced model of parkinsonism. Further studies are needed in order to assert its toxicity and mechanism of action.

## 5. Material and Methods

### 5.1. Licenses, Permits and Ethical Issues

The capture of wasp specimens was authorized by the Brazilian government agency Chico Mendes Institute for Biodiversity Conservation (ICMBio, license number 21723-1, date of issue 27 October 2009). The husbandry and manipulation of experimental animals was approved by the Ethics Committee of the University of Brasília (UnBDOC n° 85136/2013, date of issue 13 August 2013) and followed the ethical principles in animal experimentation in accordance with the Arouca law (law n° 11.794/2008).

### 5.2. Biological Material

Nests of the wasp *Parachartergus fraternus* were collected on the Darcy Ribeiro Campus of the University of Brasilia (UNB), Brasília, Distrito Federal, and immediately stored on ice until arrival at the Neuropharmacology Laboratory—UNB, where they were euthanized by freezing at −20 °C and identified.

### 5.3. Purification and Identification of the Peptide

The venom reservoirs were manually removed from female wasps, macerated in a 1:1 solution of acetonitrile and ultra-pure water and then centrifuged at 10,000 g for 3 min, at 4 °C. The supernatant was filtered on a Microcon^®^ (Millipore, Burlington, MA, USA) 3000 Da centrifugation filter and freeze-dried at the end of the process, obtaining the low-molecular-weight compounds of the venom. This extract was re-suspended 4 mg each time in 200 µL of 5% acetonitrile solution, and the components were separated by HPLC (Shimadzu Prominence, Kyoto, Japan) using a reverse phase ODS C18 column (10 µm, 250 × 10.0 mm Phenomenex^®^, Torrance, CA, USA). We used a linear gradient from 5 to 65% CH_3_CN/H_2_O/0.1% TFA at a flow of 1.5 mL/min. The absorbances were monitored at 216 and 280 nm, and the total time of chromatography was 100 min. The Fraternine peptide eluted at 50 min on a gradient of about 50% CH_3_CN/H_2_O/0.1% TFA.

The peptide fractions isolated after HPLC were submitted to mass spectrometry using a Matrix-Assisted Laser Desorption Ionization time of flight (MALDI TOF/TOF) Autoflex speed (Bruker Daltonics^®^, Bremen, Germany), to inspect molecular masses, to identify the primary amino acid sequences and to verify the degree of sample purity. Fractions were resuspended in deionized water, diluted (1:3) in a matrix of alpha-cyano-4-hydroxy-cinnamic acid (HCCA) and applied in triplicate in a MALDI plate (Bruker^®^ Massive MPT, Bremen, Germany) into a MALDI TOF/TOF UltraFlex III (Bruker Daltonics^®^, Germany). Positive reflected mode was used to determine the molecular mass of all fractions, while the amino acid sequence of the peptide in the fraction 6 was determined with the LIFT method. The mass spectra and peptide sequencing analyses were performed with the FlexAnalysis 3.0 software (Bruker Daltonics^®^, Germany).

In matters of structure elucidation of Fraternine, cysteine residues were reduced with DTT, alkylated with iodoacetamide and measured by UltraFlexIII MALDI-TOF/TOF mass spectrometer (Bruker Daltonics) in the LIFT mode. Briefly, an aliquot of 50 μg of the native peptide was reduced with 25 mM DTT in 50 mM ammonium carbonate buffer and incubated at 60 °C for 60 min under constant agitation. After reduction, the sample was alkylated with 25 mM iodoacetamide in 50 mM ammonium carbonate buffer and incubated for 40 min under the conditions described above and under light protection.

In order to distinguish the ambiguous amino acid residues, the peptide was submitted to enzymatic digestion by trypsin and GluC. Initially, immobilized trypsin TPCK (Pierce) was washed 5 times with ammonium bicarbonate buffer solution 50 mM (Pierce). Then, the peptide was incubated at 37 °C for 8 h with this enzyme and an enzyme:substrate ratio of 1:25 (v:v). Aliquots were collected at time points 0, 1, 3 and 5 h and submitted to mass spectrometry, as described previously. In relation to GluC, the alkylated sample was digested with Glu-C endopeptidase (Sigma-Aldrich, St Louis, MO, USA) for 60 min at 37 °C under constant agitation, with an enzyme:substrate ratio of 1:25 (v:v).

To analyze the matrix solution, this was prepared by weighing 5.0 mg of HCCA which was then solubilized with 250 μL of ACN, 200 μL of deionized water, and with 50 μL of an aqueous TFA solution (at 3% by volume). Then, 1 μL of the sample solution was mixed with 3 μL of the saturated matrix solution and the mixture was left to dry at room temperature (20 °C) for about 20 min. All the MS analyses were performed in a Bruker Daltonics Ultraflex III (Billerica, MA) with the external calibrations performed according to Bruker Daltonics instructions. Fragmentations for manual de novo peptide sequencing were performed by MALDI-TOF/TOF MS using the LIFT™ method. Sequencing was manually performed by using the PepSeq software (MassLynx 4.0, Waters, Milford, MA, USA).

Similarity searches were performed using blastp (http://www.ncbi.nlm.nih.gov/blast) and Fasta3 (http://www.ebi.ac.uk/fasta), with an e value cutoff set to <10^−5^ to identify putative functions. Since similarities were found, multiple sequence alignment by the Clustal omega software was performed [41].

### 5.4. Experimental Animals

Swiss male mice (*Mus musculus*) aged 5 to 7 weeks and weighing between 20 and 30 g were used in this study. All animals were maintained under controlled conditions in a 12 h light/dark cycle and at a constant temperature (23 °C) with food and water available ad libitum before and after experiments.

### 5.5. Surgical Procedure and Treatments

The surgical procedure was performed under anesthesia of the animals with ketamin and xylazine (75 mg/kg and 15 mg/kg respectively), in a stereotaxic apparatus (Insight^®^, Ribeirão Preto, Brazil). The nigro-striatal pathway was lesioned unilaterally by injecting 4 uL (40.6 ug/animal, according to Lane and Dunnet [42]) of 6-OHDA (Sigma-Aldrich). The striatal coordinates used were as follows: AP, 0.0; ML, −2.5; DV, −3.5 relative to the bregma [43]. Animals from the SHAM group had a sham surgery, in which all the surgical procedure was done in the same way but, instead of 6-OHDA, saline was injected.

In order to administer the Fraternine peptide, a guide cannula (10 mm in length) was implanted in the right lateral ventricle following the coordinates of +AP, 0.2, ML + 1.0, DV, −2.3 mm in relation to the bregma 16. The four doses of Fraternine (0.01, 0.1, 1 and 10 µg/animal) as well as saline (vehicle control) were administered via i.c.v. three times: on day 1 (one hour after surgery), day 3 and day 5 after 6-OHDA lesioning, always at the same time of day (Figure 5). Treatments were administered through the guide cannula with an injection needle (10.2 mm) in a final volume of 1 µL/animal. All the i.c.v. injections were administered with an infusion pump (Harvard Apparatus^®^, Holliston, MA, USA).

**Figure 6:**
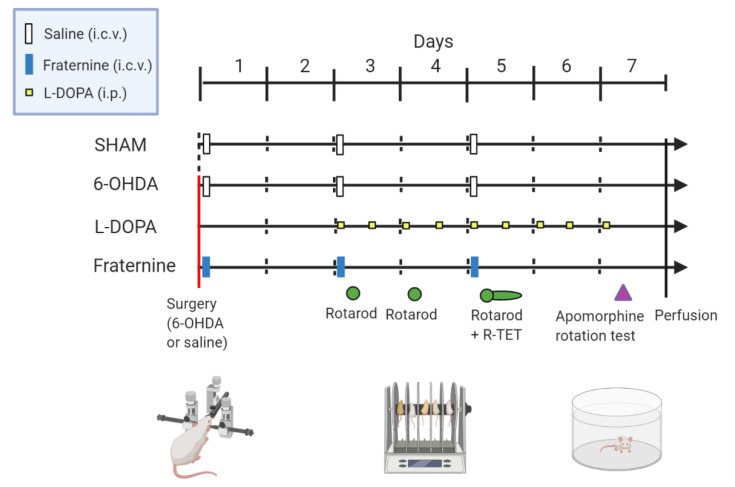
Graphical representation of the administration regimen and behavioral evaluation of animals treated with Fraternine peptide, L-DOPA/Benserazide or saline after 6-OHDA lesioning or SHAM (without lesion). On day one, animals received an intrastriatal injection of 40.6 ug of 6-OHDA (red line) or saline (dotted line) and a guide cannula was implanted in the left ventricle. Treatments was performed via i.c.v. injection for Fraternine (blue rectangle) and saline (white rectangle) and were administered twice a day via i.p. for L-DOPA/Benserazide (yellow squares). The behavioral tests consisted of rotarod motor discoordination test (Rotarod—green circle), six-hour rotarod temporal evaluation test (R-TET-green ellipse) and apomorphine-induced rotation test (purple triangle).

An additional group of animals was treated twice a day with L-DOPA methyl ester (6 mg/kg) combined with benserazide (5 mg/kg) administered via i.p. after the third day of the lesion until the seventh day, which corresponded to 9 injections of L-DOPA/BEZ. The doses were administered twice a day every 12 h. On days 3, 4 and 5, the animals were subjected to the rotarod motor discoordination test 30 min after injection of the drugs. On day 5, the animals were subjected to a 6-hour motor evaluation test and discoordination test (Figure 6).

### 5.6. Rotarod Motor Coordination Tests

The motor alterations caused by the neuronal damage induced by 6-OHDA were evaluated using two rotarod tests. The rotarod apparatus (Insight, Brazil) consists of a rotating metal cylinder, 5 cm in diameter, which rotates at a constant speed of 20 rpm. The day before 6-OHDA lesioning, all mice were pre-trained on the apparatus by placing them on the rotarod five times. Each session lasted 300 s. Only animals capable of staying on the apparatus for 300 s were subjected to the lesion (modified from Shimohama [44]). Animals were tested again in the rotarod apparatus on days 3, 4 and 5. The rotarod test consisted of placing the animals on top of the rotating cylinder and registering the length of time the animal remained on the cylinder. For treatments administered on the same day as the test, the evaluation occurred 30 min after drug administration (maximum action of L-DOPA/BEZ).

On day 5 after lesioning, motor discoordination of the animals was also observed in a test on the rotarod apparatus we called “the rotarod temporal evaluation test” or R-TET. This test consisted of evaluating the latency to fall from the rotarod apparatus at different consecutive intervals up to a maximum of 6 h: 0, 15, 30, 45, 60, 90, 120, 150, 180, 210, 240, 300 and 360 min after the last treatment on day 5. All animals, from peptide and control groups, were subjected to this test.

In addition, this test was performed on another control group (*n* = 6), which received administration of the peptide at the maximal dose (10 μg/animal) and underwent the same experimental protocol, without the 6-OHDA lesion, in order to evaluate the toxicity of the peptide that could result in motor deficits. Rotarod is a sensitive test in the detection of toxic effects of new drugs on neuromuscular coordination [45].

### 5.7. Apomorphine-Induced Turning Behavior

Apomorphine-induced turning behavior was executed following the test performed by Ungerstedt [46]. On the seventh day after 6-OHDA lesioning, animals received a subcutaneous injection of the dopaminergic agonist apomorphine (AP) (5 µg/animal-Sigma^®^, St. Louis, MO, USA). Thirty minutes after the injection, animals were placed in a transparent arena (30 cm diameter) and filmed by a video camera coupled to a computer for 15 min. The rotational behavior induced by apomorphine was analyzed as the number of contralateral rotations and was used to evaluate the severity of striatal lesioning in the animals.

### 5.8. Immunohistochemistry

After the apomorphine-induced rotation test, animals were euthanized in a CO_2_ chamber and transcardially perfused with saline and 4% formaldehyde solutions. The brains were then extracted and post-fixed in 4% formaldehyde solution for 24 h. After this period, they were stored in 30% sucrose solution (0.1 M phosphate buffer, pH 7.4) for 48 h. The brains were sectioned in coronal slices of 50 μm thickness with the aid of a vibration microtome (KD- 400, Zhejiang Jinhua Kedi Instrumental, Jinhua, China) and slices were stocked in anti-freezing solution until processing.

For the immunofluorescence assay, slices were first washed in phosphate buffered saline (PBS) solution twice for 10 min and then permeabilized with 0.8% Triton X-100 PBS for 1 hour. After permeabilization, slices were washed three times with PBS for 5 min and then incubated with a protein blocking solution (1% BSA, 10% skim milk, 0.3 M glycine, 0.1% Tween 20) for 1 hour. After another 3 PBS washes for 5 min each, they were incubated with antibody anti-tyrosine hydroxylase (Abcam^®^ ab112, Cambridge, MA, USA) 1:1000 dilution for 48 h at 4 °C and under constant stirring. After the end of incubation and appropriate washes, the brain sections were incubated with the secondary antibody anti-rabbit IgG H&L conjugated with Alexa Fluor 488 (Abcam^®^ ab150077) at a concentration of 1:400 (diluted in PBS + 1% BSA) at room temperature with constant stirring for 3 h protected from light. Secondary antibody was washed off, and slices were mounted on glass slides with fluorescent mounting medium with DAPI (Sigma^®^). Photomicrographs were acquired using a digital camera coupled to an epifluorescent microscope (Leica DM 2000, Wetzlar, Germany). Sections were photographed with 200× magnification in both striatum and *Substantia Nigra*. The cytoplasm of TH-reactive neurons was observed as green, while the nuclei of all cells were stained with DAPI and observed as blue.

In order to evaluate neuronal depletion at the SN, TH-reactive cells were directly counted. Since we used a hemi-parkinsonian model, the hemisphere which did not receive 6-OHDA (right hemisphere) was considered as a health control, in order to evaluate and compare the two sides of the brain. For each subject of the group, three slices of the SN region (*n* = 3 to 8) were used. Results were expressed as a percentage of surviving neurons from the lesioned side when compared to the contralateral (unlesioned) side.

### 5.9. Statistical Analysis

Data from the apomorphine-induced rotation test, motor coordination test and surviving neuron evaluation were submitted to two-way ANOVA followed by Bonferroni multiple comparisons. Data obtained from the R-TET were calculated with linear mixed model with animals as random effect to control for repeated measures. Permanence times were normalized with Box-Cox transformation (λ = −0.3) to attain normality. Significant differences between groups were considered when *p* < 0.05. Box-Cox transformation and mixed linear model was performed on R program (version 3.0, R Foundation, Vienna, Austria) and the remaining analyses were performed on GraphPad Prism^®^ 7.0 (San Diego, CA, USA).

## 6. Patents

This work has resulted in the patent of code BR 10 2018 008420 8 deposited in the INPI in Brazil, which relates to a synthetic peptide inspired by Fraternine.

## Figures and Tables

**Table 1:** Fraction, molecular mass, name, sequence and known effects of the peptides from *P. fraternus* venom.

Fraction ^a^	MM	Peptide	Sequence	Effects
3	1055.6	unknown	-^b^	-
4	1358.8	unknown	-^b^	-
5	1209.9	protonectin	ILGTILGLLKGL-NH_2_	chemotactic peptide, antimicrobial, anticancer activity on breast cancer cells [26,27]
6	2748.5	Fraternine	I/LSFQQVKEQVCKVEAQI/LGQQI/LPFC-NH_2_	Neuroprotective effect (this study)
7	1566.9	Agelaia-MP	INWLKLGKAIIDAL–NH_2_	broad-spectrum action against microorganisms, inhibitory effects against tumor proliferation, antinociceptive and stimulating serotonin release from platelets and mast cell degranulation [25,26,28]

^a^ The two initial fractions are formed by biogenic amines and neurotransmitters, therefore not peptides and not used in this study. ^b^ Fractions with no effect on the animal model were not sequenced.

**Table 2:** Theoretical and experimental masses of the fragments to obtain the structural elucidation of Fraternine.

Process	Theoretical Mass [M + H]^+^	Experimental Mass [M + H]^+^	Figure	Sequence
Red-Alkylation	2864.59	2864.6	Figure S3a,b	I/LSFQQVKEKVC (Acm)KVEAKI/LGKKI/LPFC (Acm)
GluC	-	-	Figure S4a	MS spectrum after 1 h
	-	-	Figure S4b	MS spectrum after 1.5 h
			Figure S4c	MS spectrum after 3 h
			Figure S4d	MS spectrum after 4.5 h
			Figure S4e	MS spectrum after 5.5 h
GluC	978.52	978.6	Figure S5	I/LSFQQVKE
Trypsin	-	-	Figure S6a	MS spectrum after 1 h
	-	-	Figure S6b	MS spectrum after 3 h
	-	-	Figure S6c	MS spectrum after 5.5 h
Trypsin	849.48	849.5	Figure S7a	I/LSFQQVK
	1106.62	1106.6	Figure S7b	I/LSFQQVKEK
	1493.81	1493.8	Figure S7c	I/LSFQQVKEKVC(Acm)K
	1921.06	1921.0	Figure S6a *	I/LSFQQVKEKVC(Acm)KVEAK
	2219.26	2219.2	Figure S7d	I/LSFQQVKEKVC(Acm)KVEAKI/LGK
	2347.37	2347.3	Figure S6a *	I/LSFQQVKEKVC(Acm)KVEAKI/LGKK
	2864.59	2864.5	Figure S7e	I/LSFQQVKEKVC(Acm)KVEAKI/LGKKI/LPFC(Acm)

* Ion detected with fragment in MS spectrum.

## Supplementary Materials

The following are available online at https://www.mdpi.com/2072-6651/12/9/550/s1, Figure S1: Molecular mass determination of Fraternine. Figure S2: MS/MS sequencing of the ion [M + H]^+^ *=* 2478.5. Figure S3: Mass analysis of the peptide after reduction and alkylation processes. Figure S4: Mass analysis of the peptide after reduction and alkylation processes and enzymatic digestion with GluC. Figure S5: MS/MS sequencing of the ion [M + H]^+^ = 978.6. Figure S6: Mass analysis of the peptide after reduction and alkylation processes and enzymatic digestion with Trypsin. Figure S7. MS/MS sequencing of the ions after reduction and alkylation processes and enzymatic digestion with Trypsin. Figure S8. Toxicity test on the rota-rod R-TET assay
