# Supplementary material for: Fraternine, a Novel Wasp Peptide, Protects against Motor Impairments in 6-OHDA Model of Parkinsonism
Source: Toxins (Basel). 2020 Aug 27;12(9):550. doi: 10.3390/toxins12090550 (PMC7551070; doi:10.3390/toxins12090550)
Supplement: Supplementary file 1 [file toxins-12-00550-s001.pdf]

Supplementary Materials: Fraternine, a Novel Wasp Peptide, Protects against Motor Impairments in 6-OHDA Model of Parkinsonism

Andréia Mayer Biolchi, Danilo Gustavo Rodrigues de Oliveira, Henrique de Oliveira Amaral, Gabriel Avohay Alves Campos, Jacqueline Coimbra Gonçalves, Adolfo Carlos Barros de Souza, Marcos Robalinho Lima, Luciano Paulino Silva and Márcia Renata Mortari

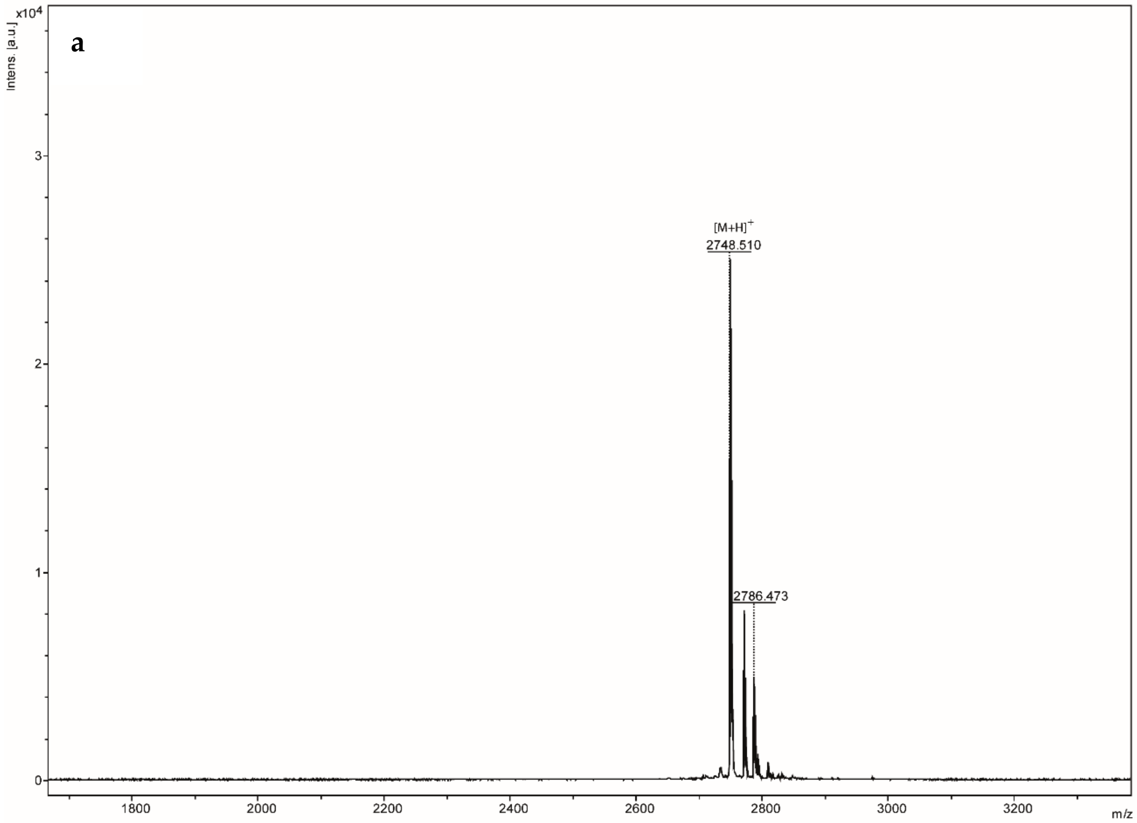

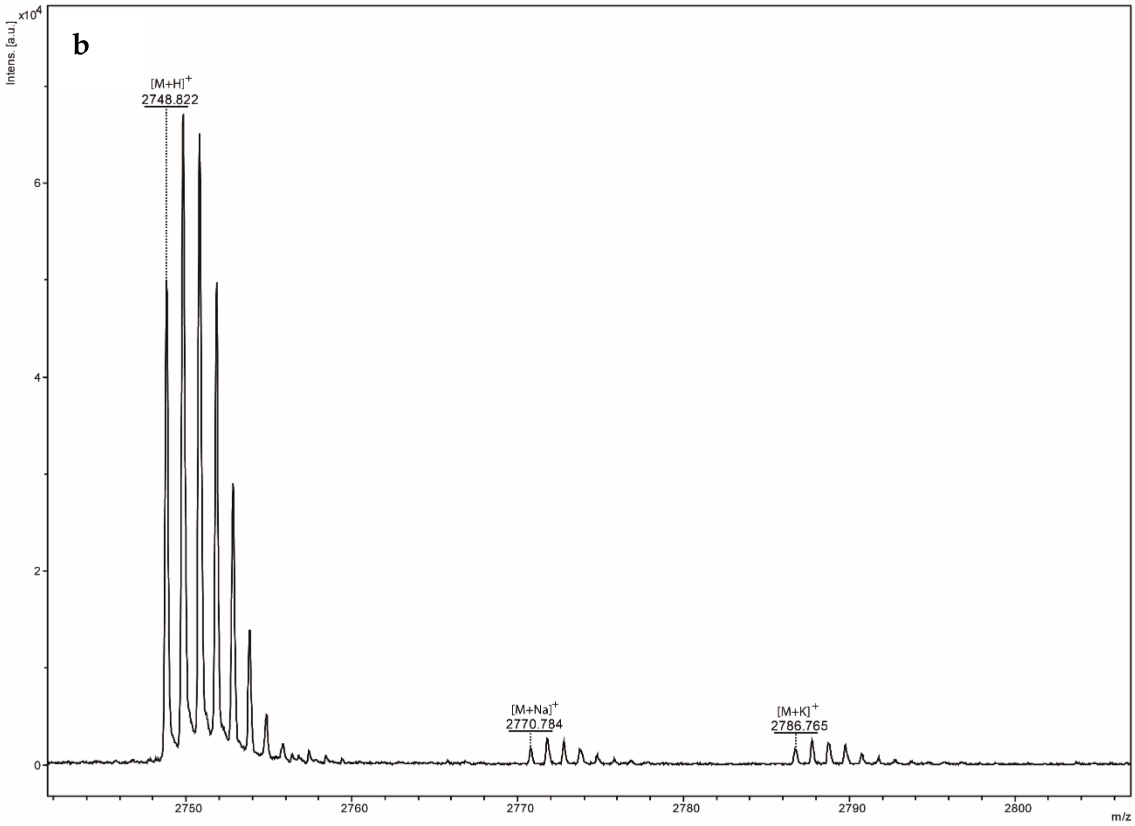

Figure S1. Molecular mass determination of Fraternine. (**a**) Mass spectrometry analysis of peptide obtained in the reflected mode showing mass of $[M + H]^+ = 2748.5$. (**b**) Mass spectrometry analysis showed the adducts with Na^+ and K^+.

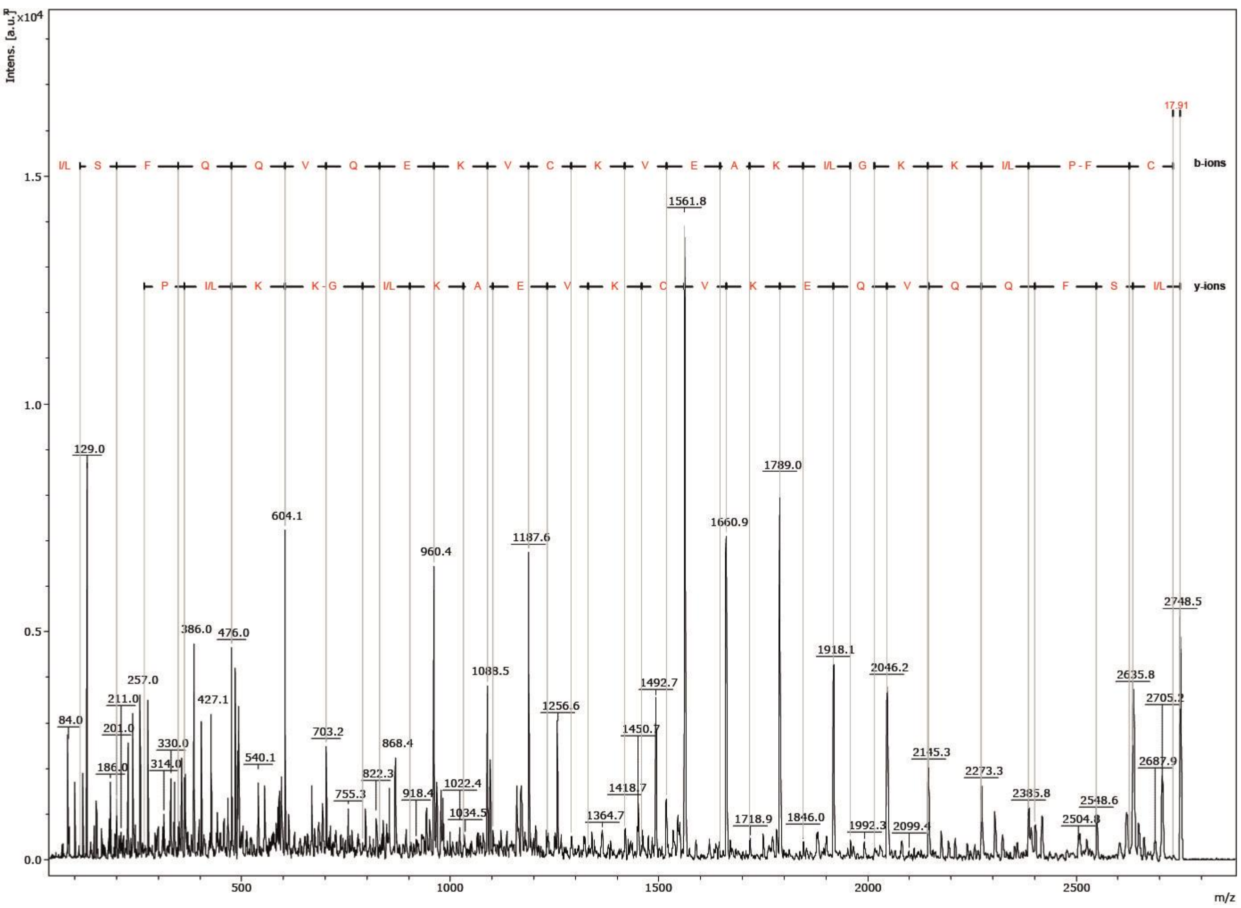

Figure S2. MS/MS sequencing of the ion [M + H]+ = 2478.5. The y-ion and the b-ion series are shown. Sequencing de novo obtained from the peptide Fraternine. Performed by MALDI TOF/TOF in LIFT mode.

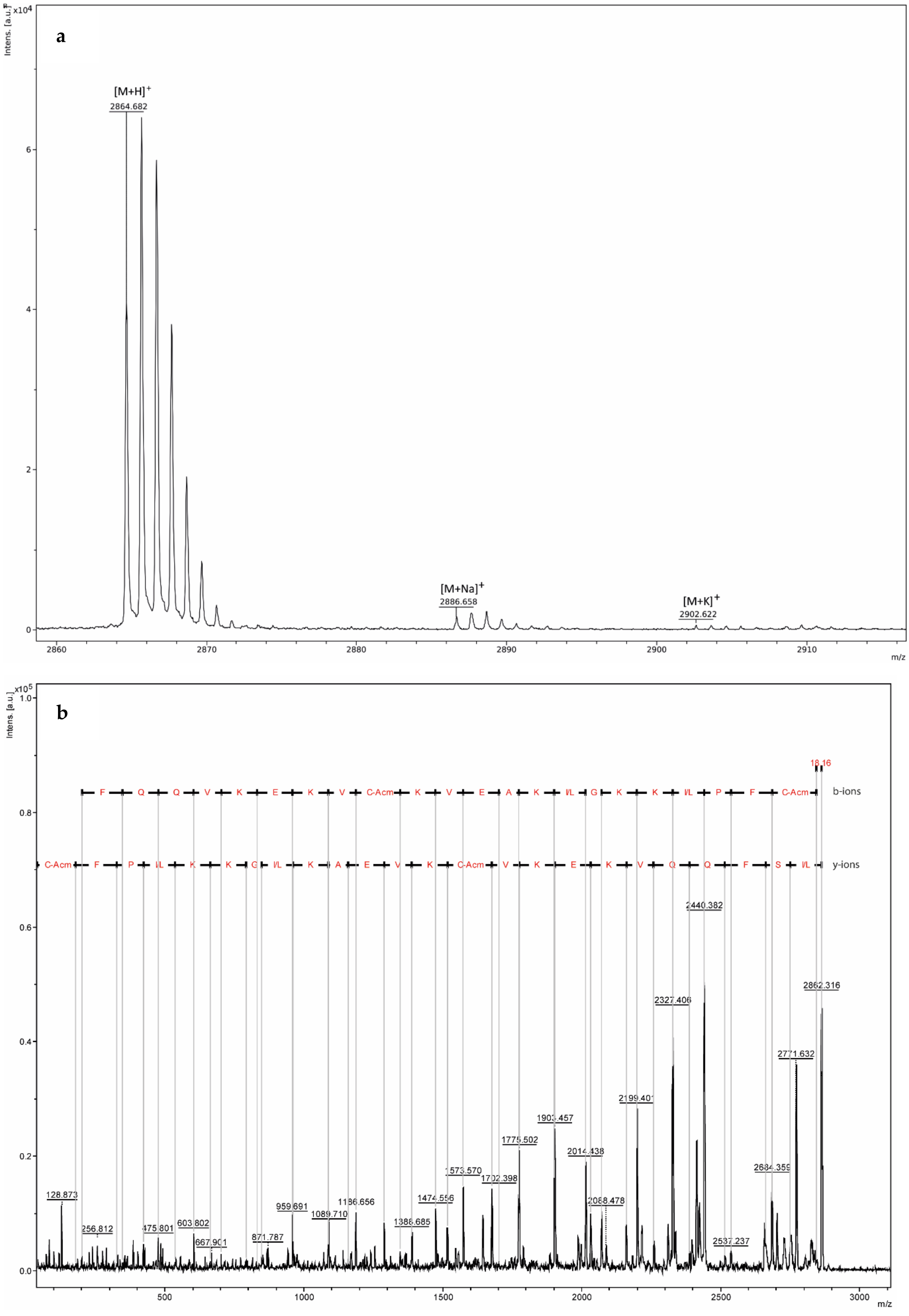

Figure S3. Mass analysis of the peptide after reduction and alkylation processes. (**a**) Mass spectrometry analysis of peptide obtained in the reflected mode showing mass of $[M + H]^+ = 2864.6$. (**b**) MS/MS sequencing of the ion $[M + H]^+ = 2864.6$. The y-ion and the b-ion series are shown.

Sequencing de novo obtained from the ion [M + H]$^+$ = 2864.6. Performed by MALDI TOF/TOF in LIFT mode.

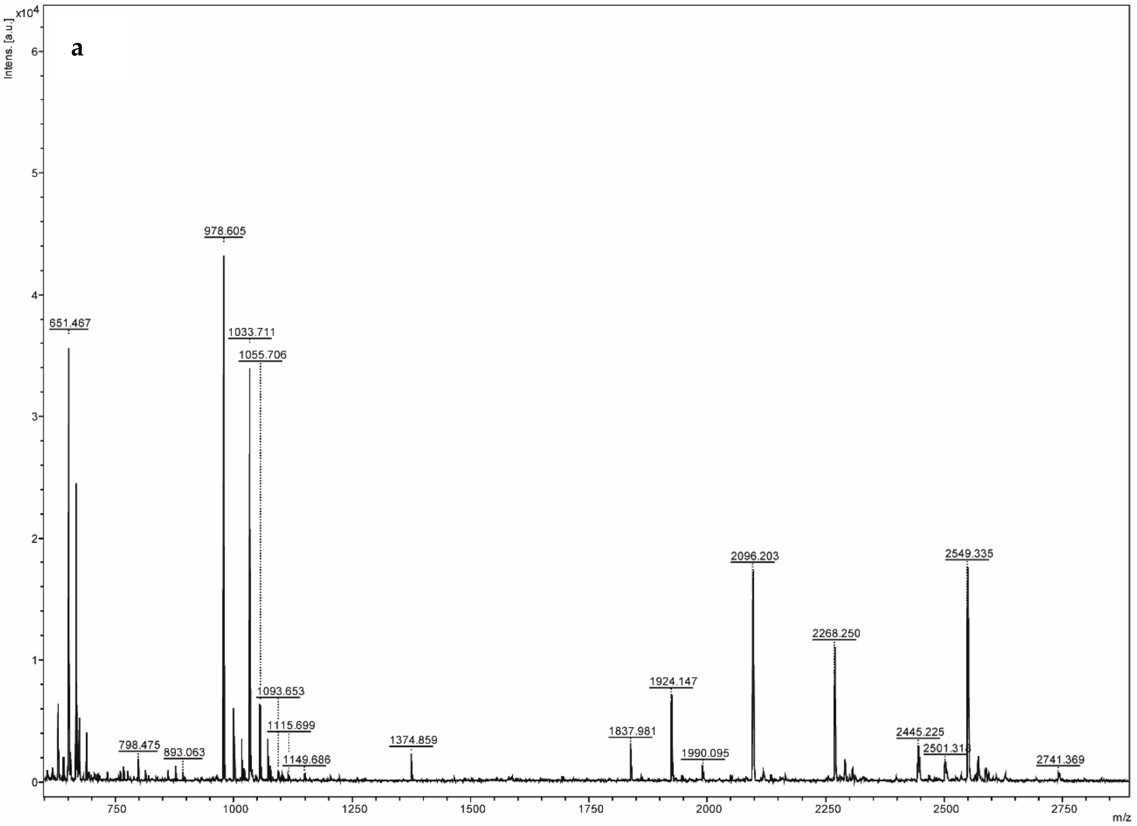

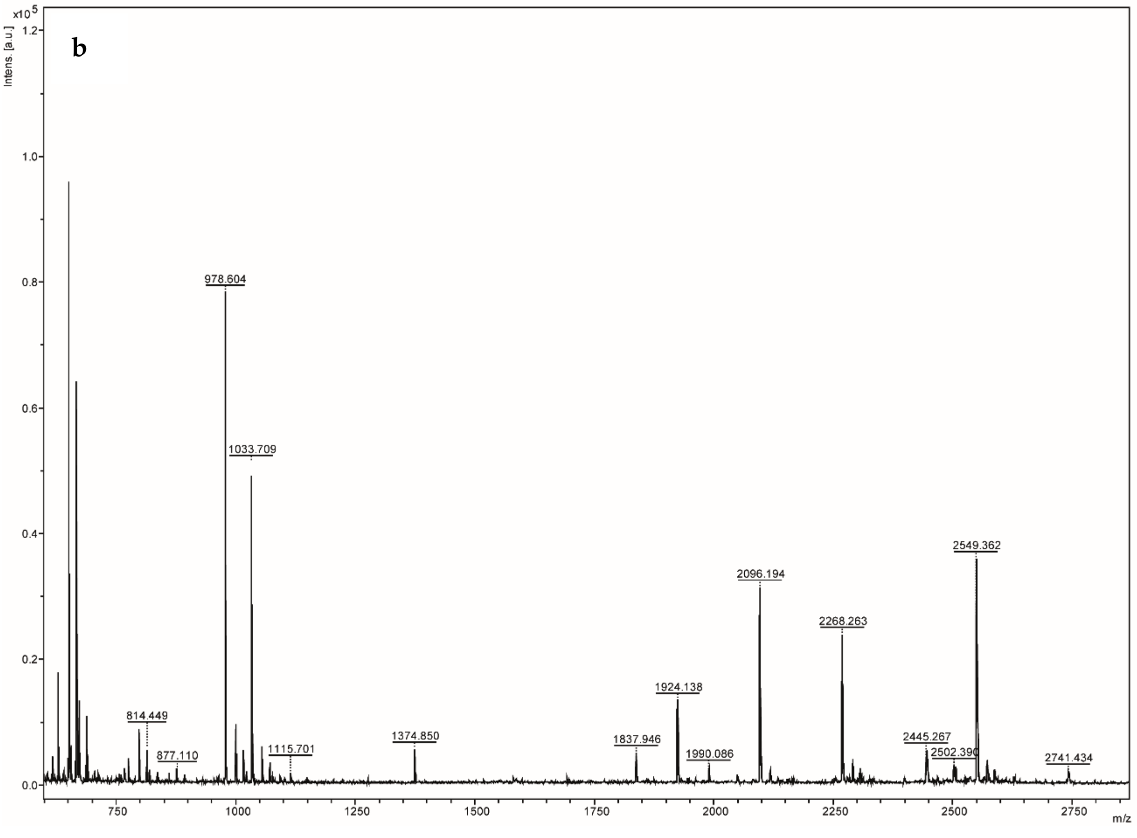

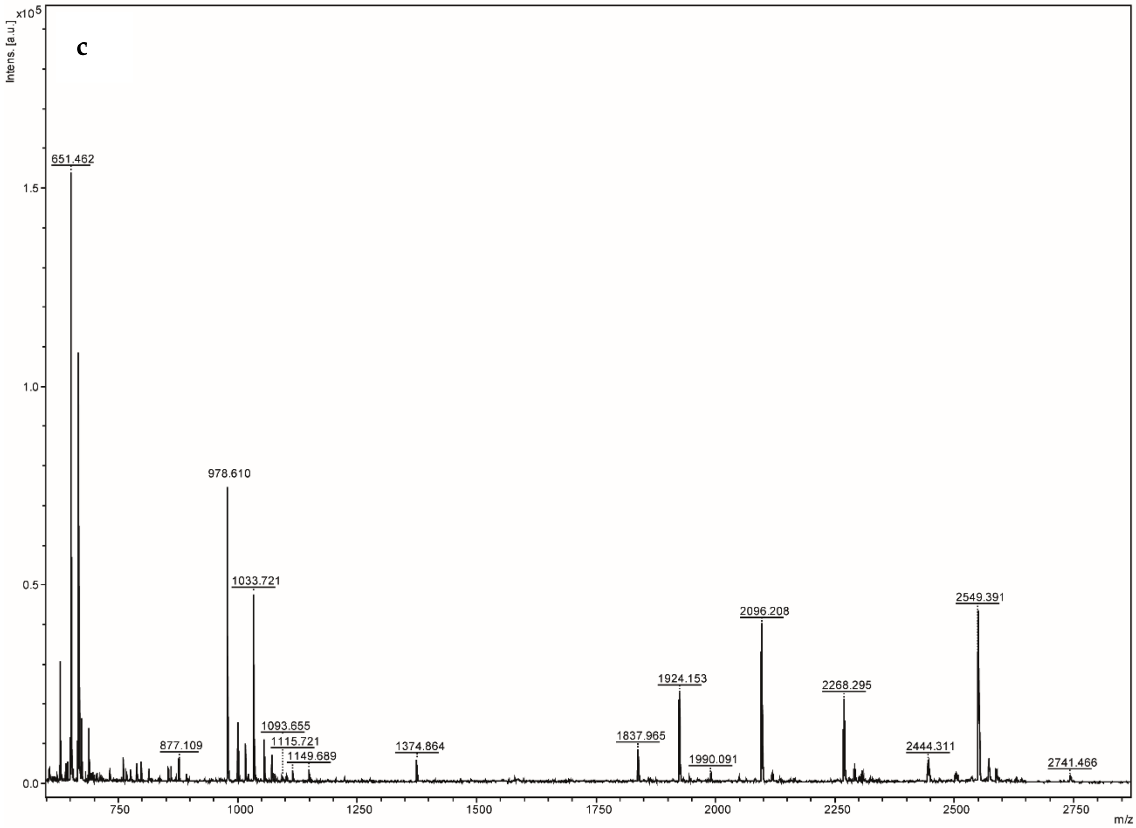
c
x10^5
Intens. [a.u.]
651.462
978.610
1033.721
877.109
1093.655
1115.721
1149.689
1374.864
1837.965
1924.153
1990.091
2096.208
2268.295
2444.311
2549.391
2741.466
m/z

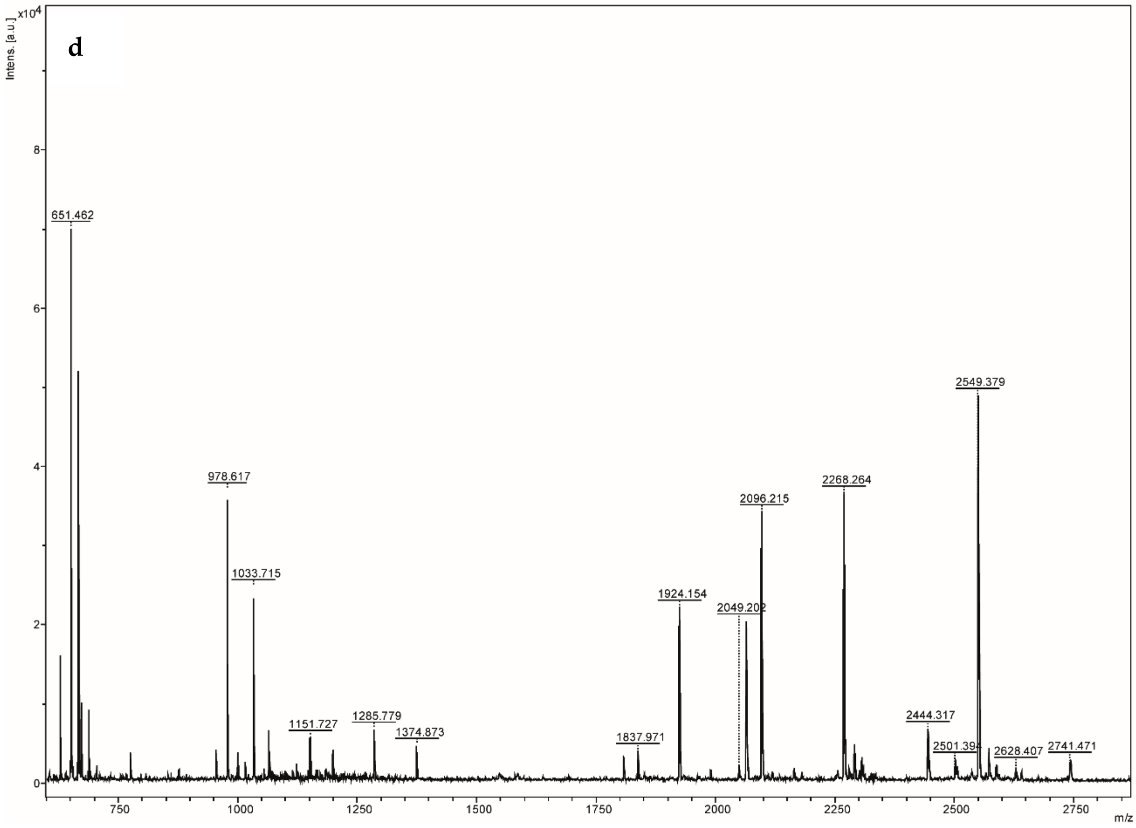
d
x10^4
Intens. [a.u.]
651.462
978.617
1033.715
1151.727
1285.779
1374.873
1837.971
1924.154
2049.202
2096.215
2268.264
2444.317
2501.394
2549.379
2628.407
2741.471
m/z

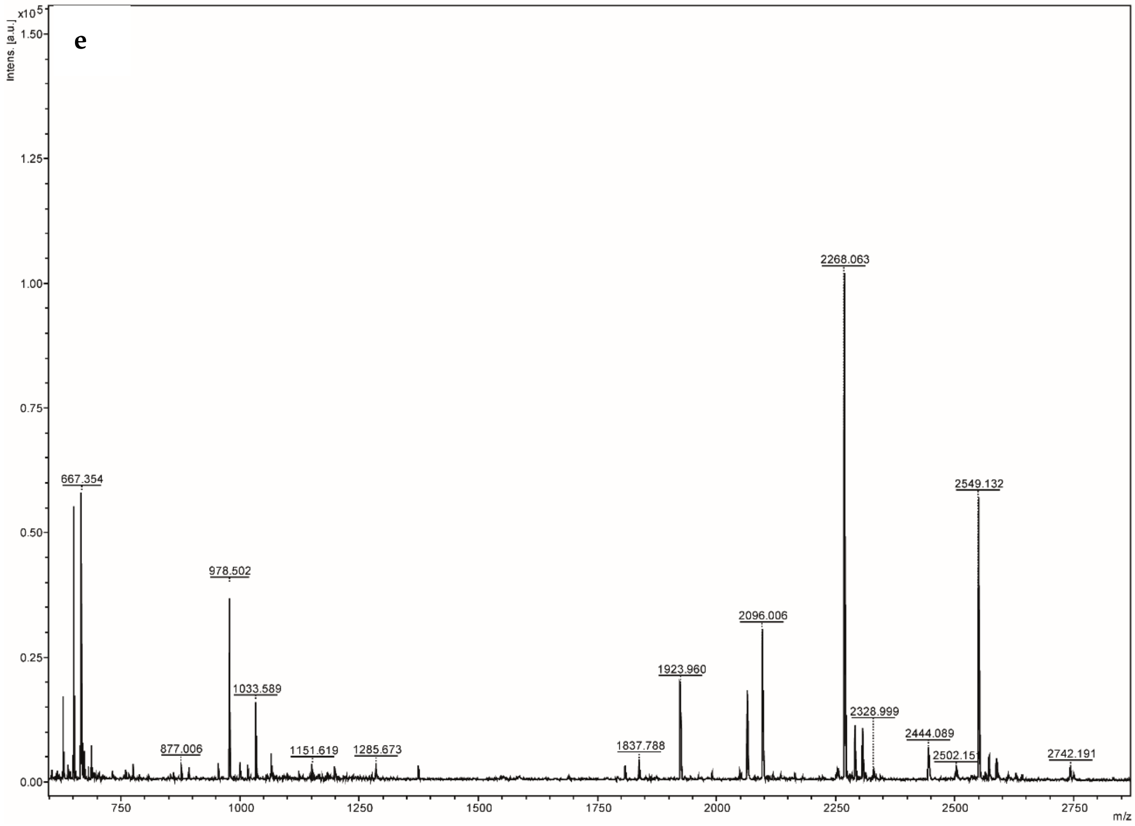

Figure S4. Mass analysis of the peptide after reduction and alkylation processes and enzymatic digestion with GluC. (**a**) Mass spectrum after 1 h of addition of GluC. (**b**) Mass spectrum after 1.5 h of addition of GluC. (**c**) Mass spectrum after 3 h of addition of GluC. (**d**) Mass spectrum after 4.5 h of addition of GluC. (**e**) Mass spectrum after 5.5 h of addition of GluC.

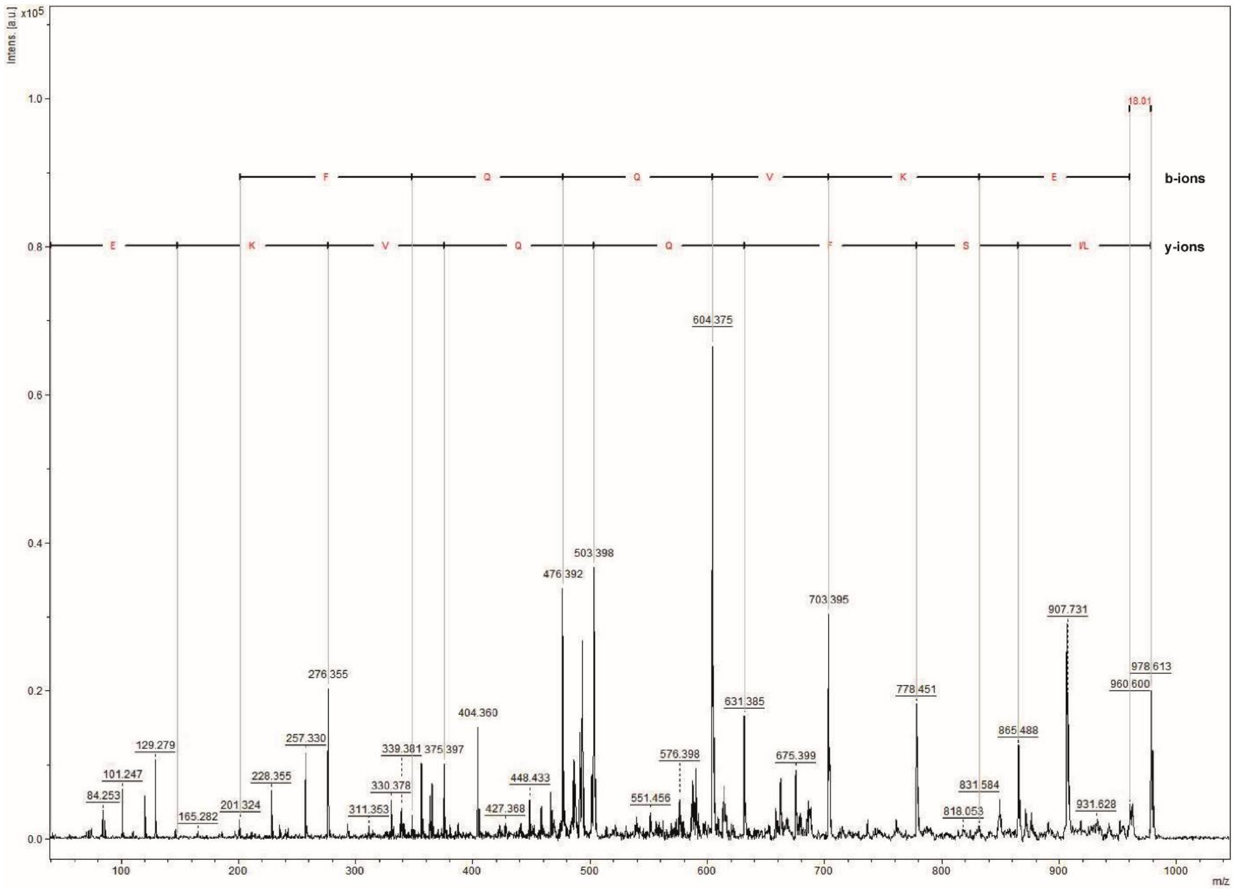

Figure S5. MS/MS sequencing of the ion [M + H]$^+$ = 978.6. The y-ion and the b-ion series are shown. Sequencing de novo obtained from the fragment after GluC digestion. Performed by MALDI TOF/TOF in LIFT mode.

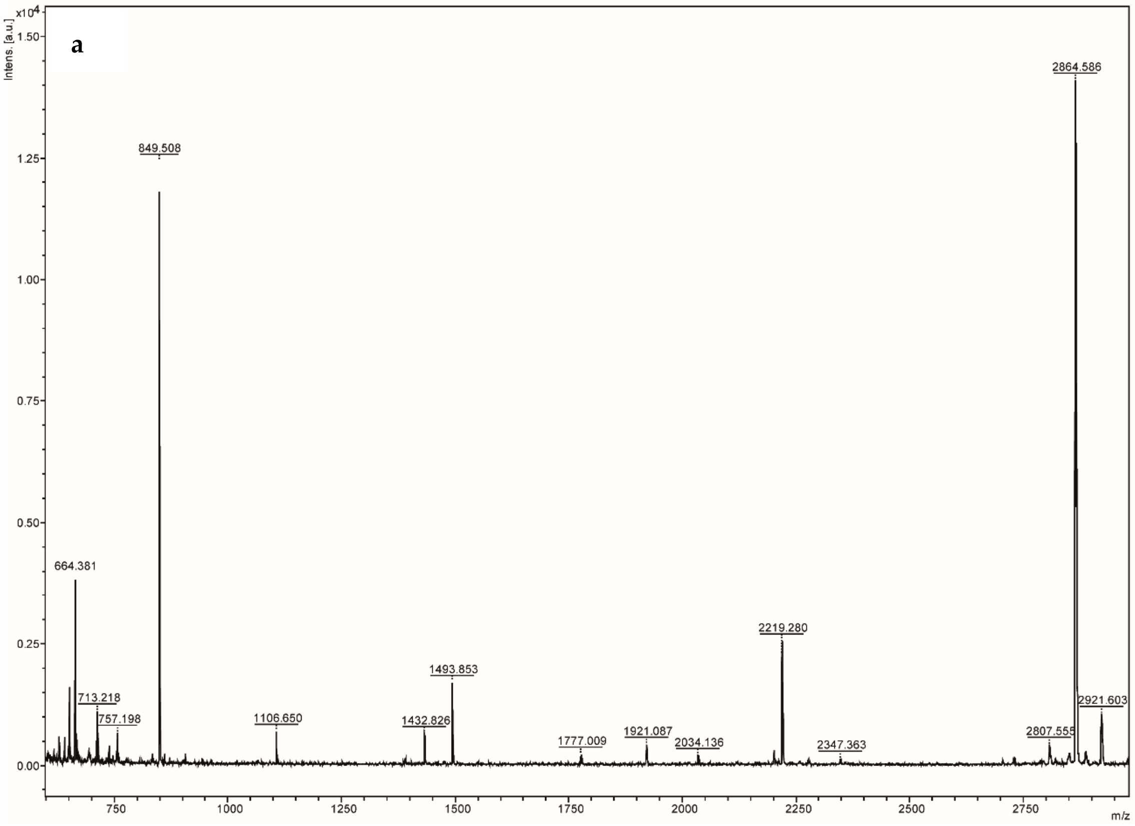
a
x10^4
Intens. [a.u.]
2864.586
849.508
664.381
2219.280
1493.853
713.218
757.198
1106.650
1432.826
1777.009
1921.087
2034.136
2347.363
2807.555
2921.603
m/z

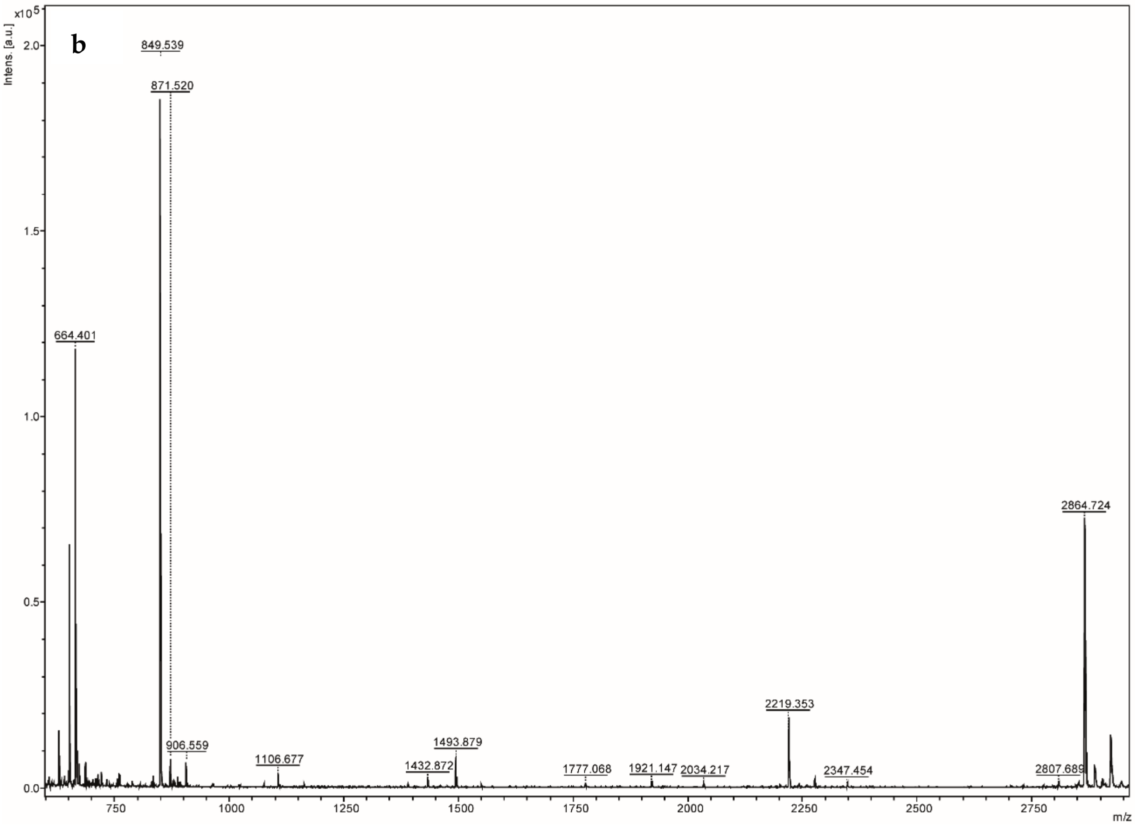
b
x10^5
Intens. [a.u.]
849.539
871.520
664.401
2864.724
2219.353
906.559
1106.677
1432.872
1493.879
1777.068
1921.147
2034.217
2347.454
2807.689
m/z

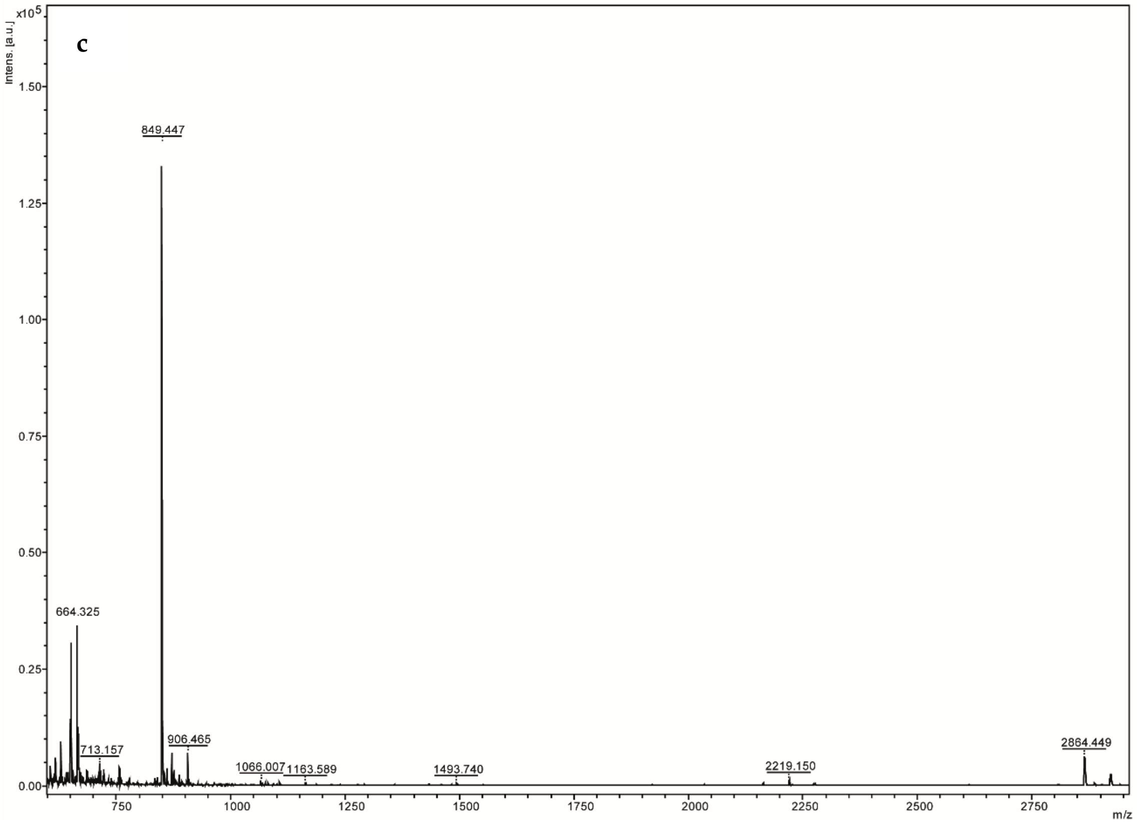

Figure S6. Mass analysis of the peptide after reduction and alkylation processes and enzymatic digestion with Trypsin. (**a**) Mass spectrum after 1 h of addition of Trypsin. (**b**) Mass spectrum after 3 h of addition of Trypsin. (**c**) Mass spectrum after 5.5 h of addition of Trypsin.

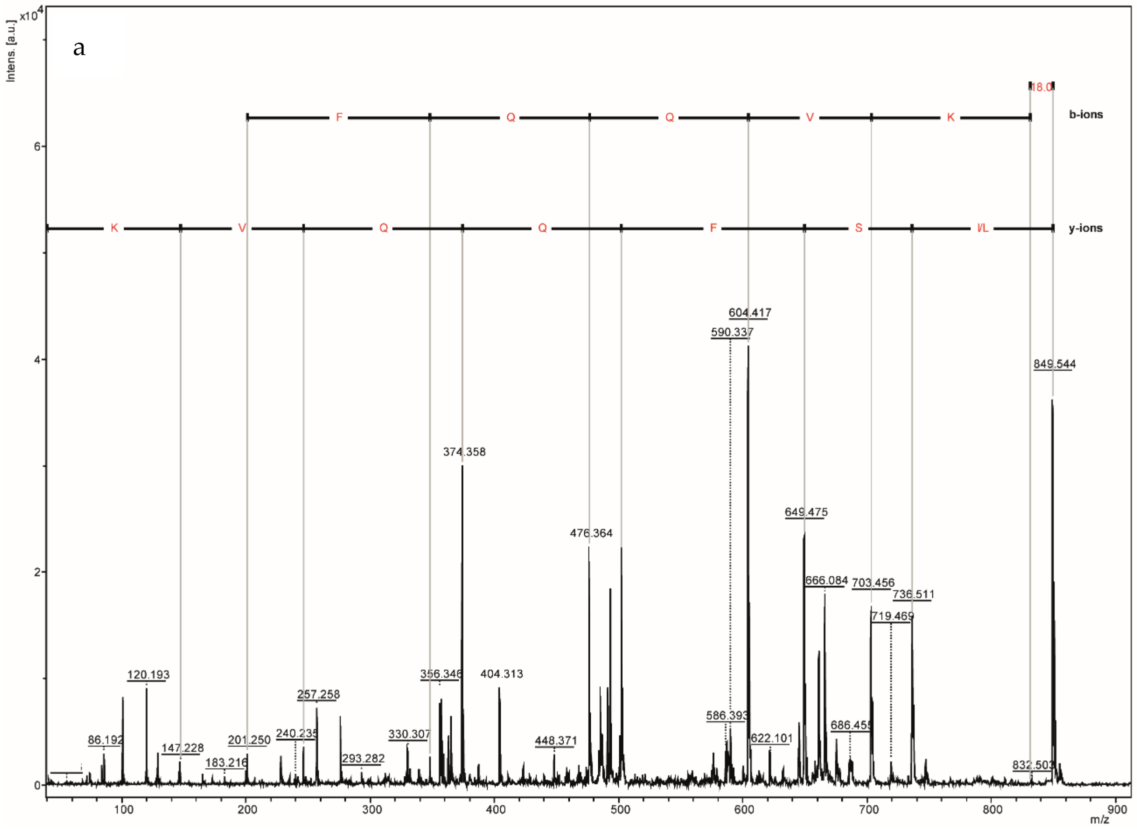
a
Intens. [a.u.]
x10^4
b-ions
F Q Q V K
18.0
y-ions
K V Q Q F S I/L
604.417
590.337
849.544
374.358
476.364
649.475
666.084
703.456
736.511
719.469
120.193
356.346
404.313
257.258
586.393
86.192
147.228
201.250
240.235
330.307
448.371
622.101
686.455
183.216
293.282
832.503
m/z

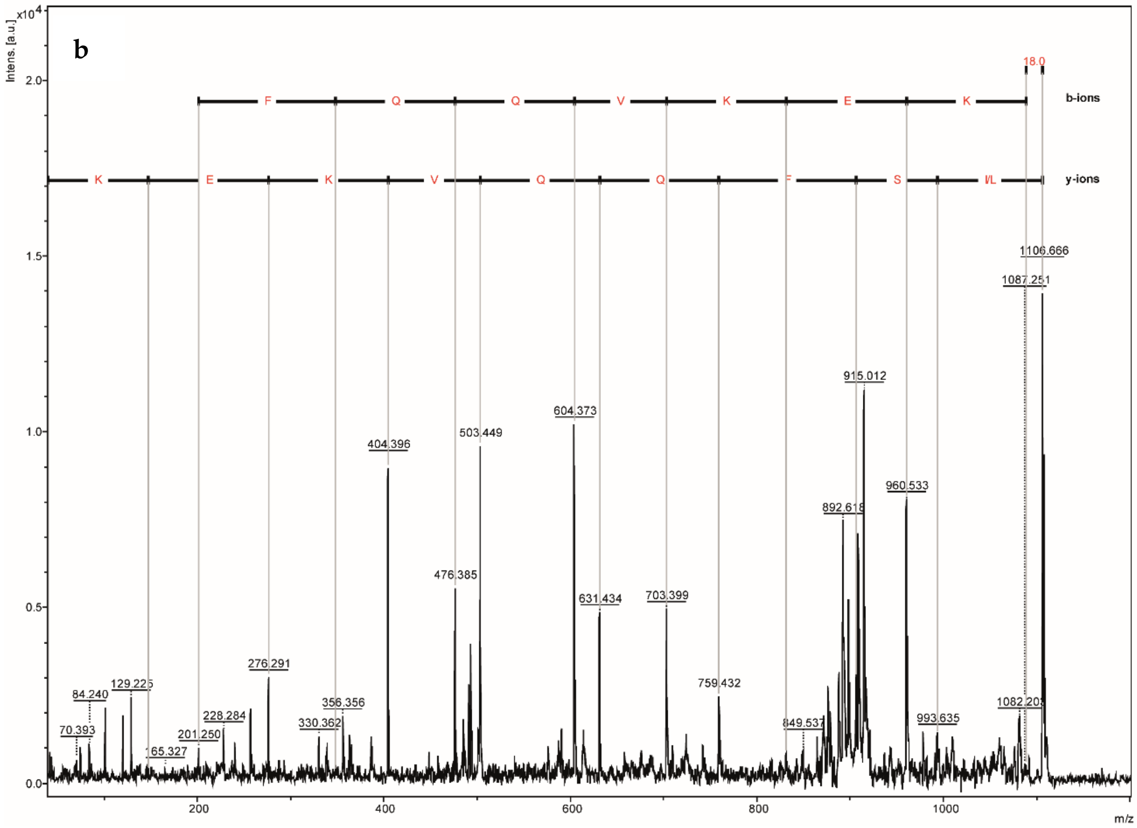
b
Intens. [a.u.]
x10^4
18.0
b-ions
F Q Q V K E K
y-ions
K E K V Q Q F S I/L
1106.666
1087.251
915.012
604.373
503.449
404.396
960.533
892.618
476.385
631.434
703.399
276.291
759.432
129.225
84.240
356.356
1082.203
228.284
330.362
849.537
993.635
70.393
201.250
165.327
m/z

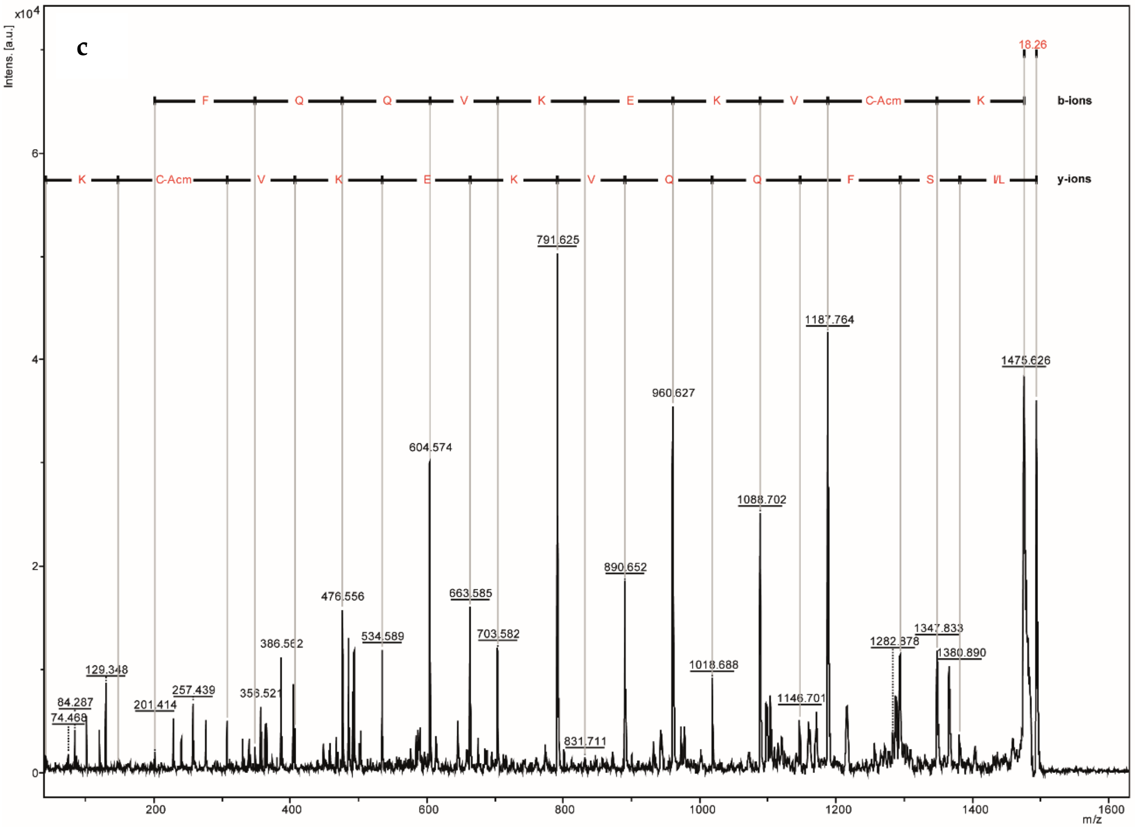
c
x10^4
Intens. [a.u.]
18.26
F Q Q V K E K V C-Acm K
b-ions
K C-Acm V K E K V Q Q F S I/L
y-ions
791.625
1187.764
1475.626
960.627
604.574
1088.702
890.652
476.556
663.585
386.562
534.589
703.582
1282.378
1347.833
1380.890
129.348
1018.688
84.287
201.414
257.439
356.521
74.468
1146.701
831.711
m/z

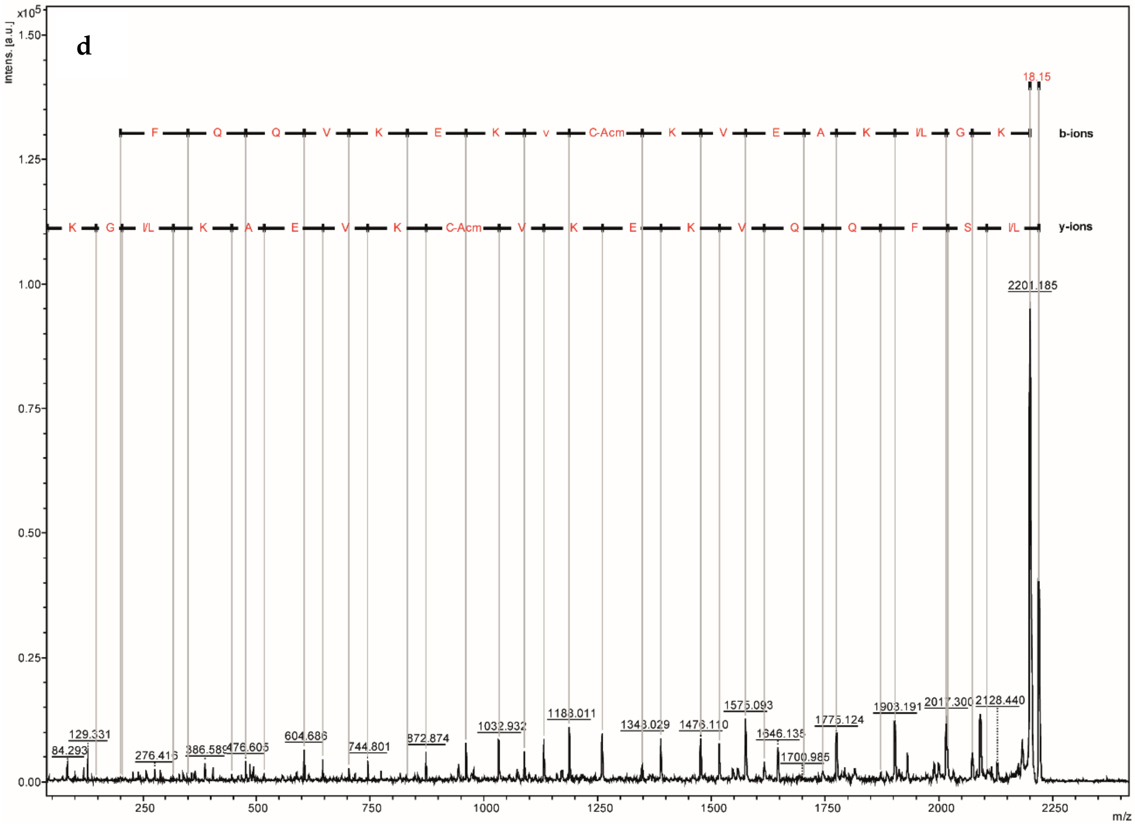
d
x10^5
Intens. [a.u.]
18.15
F Q Q V K E K v C-Acm K V E A K I/L G K
b-ions
K G I/L K A E V K C-Acm V K E K V Q Q F S I/L
y-ions
2201.185
129.331
84.293
276.416
386.589
476.605
604.686
744.801
872.874
1032.932
1183.011
1348.029
1476.110
1575.093
1646.135
1700.985
1775.124
1903.191
2017.300
2128.440
m/z

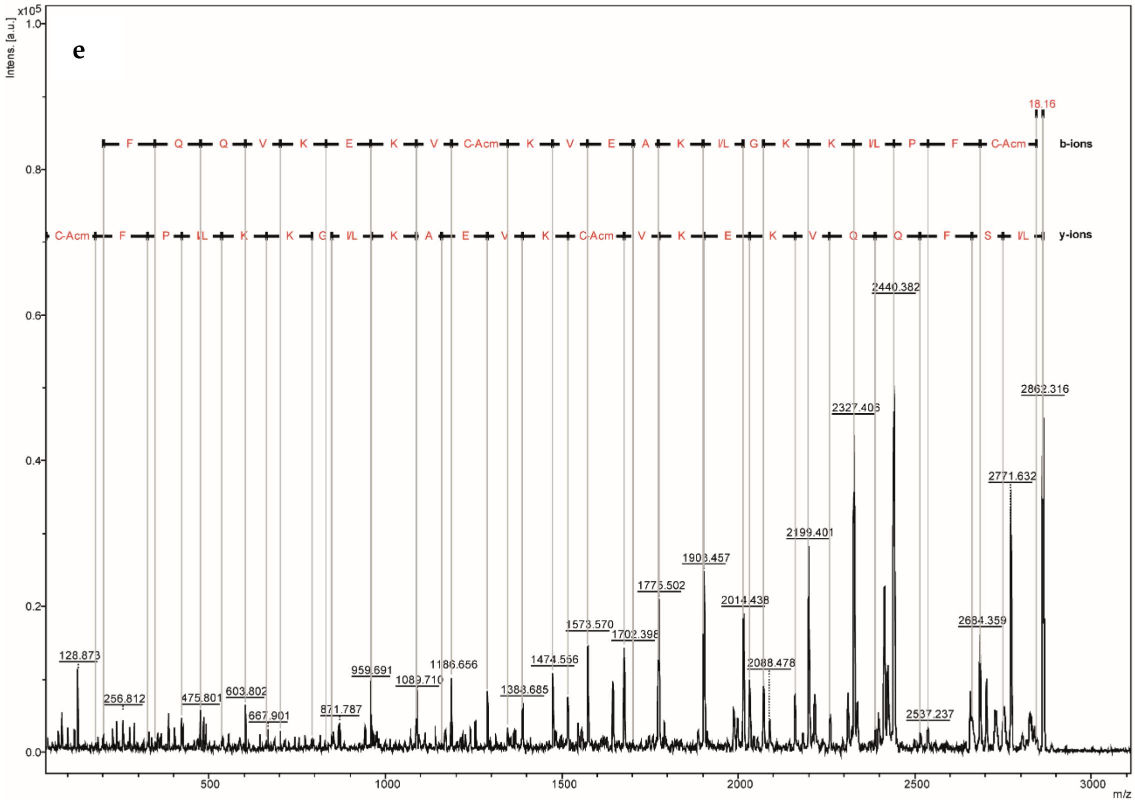

Figure S7. MS/MS sequencing of the ions after reduction and alkylation processes and enzymatic digestion with Trypsin. The y-ion and the b-ion series are shown. Sequencing de novo obtained from the fragment after GluC digestion. Performed by MALDI TOF/TOF in LIFT mode. (**a**) MS/MS sequencing of the ion $[M + H]^+ = 849.5$. (**b**) MS/MS sequencing of the ion $[M + H]^+ = 1106.6$. (**c**) MS/MS sequencing of the ion $[M + H]^+ = 1493.8$. (**d**) MS/MS sequencing of the ion $[M + H]^+ = 2219.2$. (**e**) MS/MS sequencing of the ion $[M + H]^+ = 2864.5$.

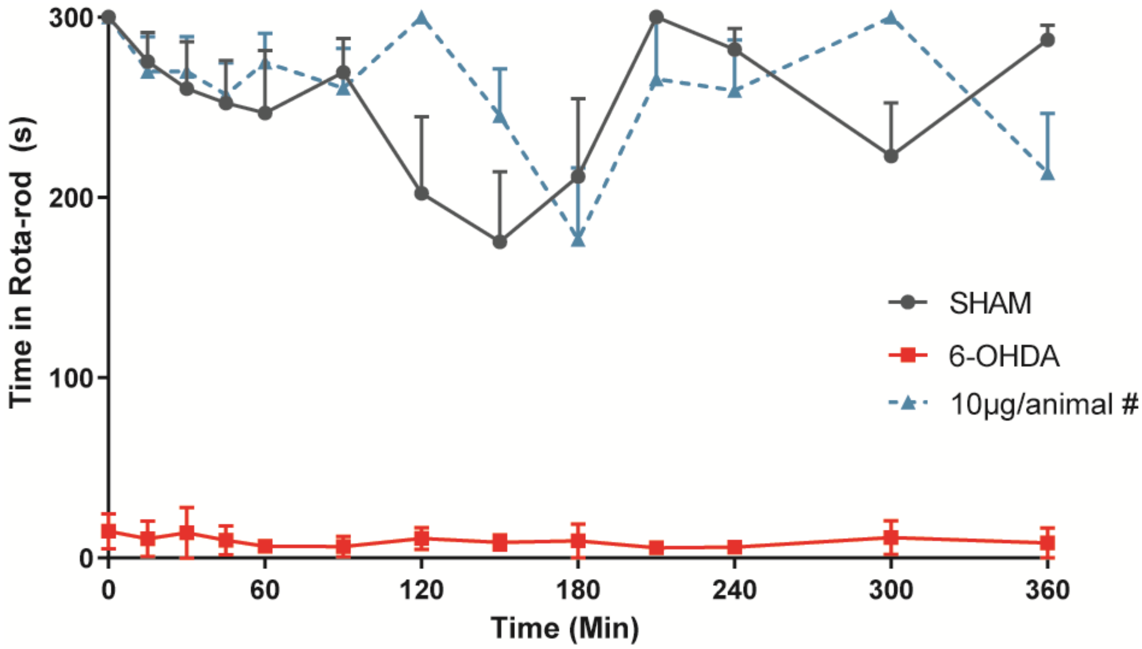

Figure S8. Toxicity test on the rota-rod R-TET assay. Latency to fall on the rota-rod apparatus was evaluated in SHAM animals ($n = 9$), 6-OHDA lesioned ($n = 7$) and animals receiving 10 µg of fraternine ($n = 8$) during a total of six hours to see if the peptide caused motor alterations in mice that prevented it to walk freely. Symbol # represents significative difference from the 6-OHDA group.
